# Individual thalamic inhibitory interneurons are functionally specialized toward distinct visual features

**DOI:** 10.1016/j.neuron.2024.06.001

**Published:** 2024-08-21

**Authors:** Fiona E. Müllner, Botond Roska

**Affiliations:** 1 Institute of Molecular and Clinical Ophthalmology Basel, 4031 Basel, Switzerland; 2 Department of Ophthalmology, University of Basel, 4031 Basel, Switzerland; 3 Friedrich Miescher Institute for Biomedical Research, 4056 Basel, Switzerland

**Keywords:** LGN, dorsolateral geniculate nucleus, vision, inhibition, inhibitory interneurons, dendrites, retina, sensory integration, attention, rabies tracing

## Abstract

Inhibitory interneurons in the dorsolateral geniculate nucleus (dLGN) are situated at the first central synapse of the image-forming visual pathway, but little is known about their function. Given their anatomy, they are expected to be multiplexors, integrating many different retinal channels along their dendrites. Here, using targeted single-cell-initiated rabies tracing, we found that mouse dLGN interneurons exhibit a degree of retinal input specialization similar to thalamocortical neurons. Some are anatomically highly specialized, for example, toward motion-selective information. Two-photon calcium imaging performed *in vivo* revealed that interneurons are also functionally specialized. In mice lacking retinal horizontal direction selectivity, horizontal direction selectivity is reduced in interneurons, suggesting a causal link between input and functional specialization. Functional specialization is not only present at interneuron somata but also extends into their dendrites. Altogether, inhibitory interneurons globally display distinct visual features which reflect their retinal input specialization and are ideally suited to perform feature-selective inhibition.

## Highlights

• dLGN interneurons have similar degrees of input specialization as thalamocortical neurons
• dLGN interneurons are functionally specialized *in vivo*, encoding diverse visual features
• Visual feature selectivity of dLGN interneurons is causally linked to their retinal input
• Visual feature selectivity of dLGN interneurons is similar in somata and dendrites

## Graphical abstract

**Figure:**
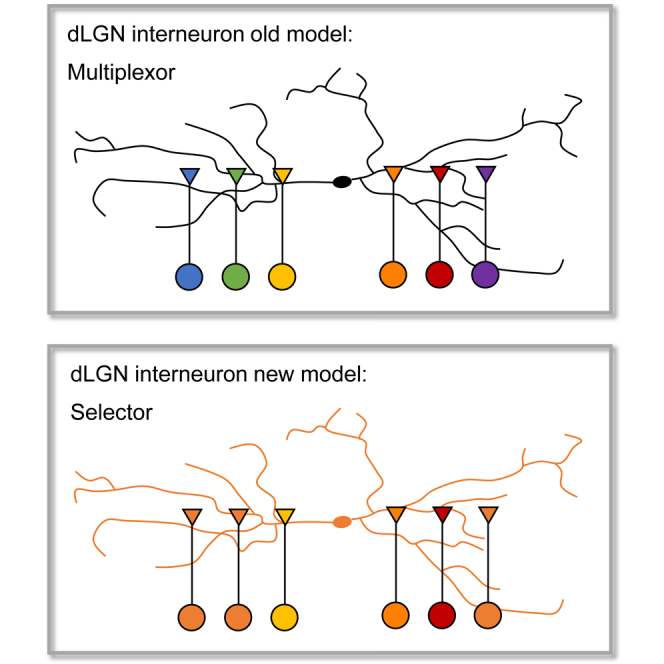

## Teaser

Müllner and Roska study inhibitory interneurons in the primary visual thalamus (dLGN). dLGN interneurons had been considered “multiplexors” that receive many independent retinal inputs. Müllner and Roska instead find that dLGN interneurons act as “selectors” with individual specialization toward subsets of retinal inputs that determine their cell-wide visual feature selectivity.

## Introduction

All sensory systems, except for smell, pass through the thalamus as information flows from the sensory periphery to the cortex. How sensory information is modified within the thalamus and the function of the thalamus in sensory processing are key and open questions in neuroscience. The dorsolateral geniculate nucleus (dLGN) is the primary visual thalamic nucleus necessary for image perception. Retinal ganglion cells (RGCs), as the output neurons of the retina, synapse onto excitatory thalamocortical neurons in the dLGN (TCNs), which in turn project to the primary visual cortex. The dLGN also contains GABAergic inhibitory interneurons that receive inputs from RGCs and synapse onto TCNs.^1^ The interneurons are ideally suited to influence visual information at this early stage,^2^^,^^3^ but little is known about their *in vivo* function or how they process retinal information.

The retina of vertebrates extracts many features from the visual scene and sends these features to the dLGN via the axons of distinct RGC types. Mice have 46 transcriptomic RGC types,^4^ each of which has characteristic morphological and electrophysiological properties.^5^ Which visual filter a specific RGC type applies (the “retinal channel”) is determined by the specific bipolar and amacrine cell input it receives^6^ and its electrophysiological properties.^7^ Note that for clarity, throughout the paper we use the term “retinal channel” to describe the visual features represented by RGCs and the term “visual feature” to describe the visual features represented by dLGN neurons. The axons of bipolar cells and the processes of amacrine cells that provide input to the dendrites of RGCs are organized in layers within the inner plexiform layer of the retina.^8^ Accordingly, the dendrites of each RGC type display a stereotypical stratification pattern within the inner plexiform layer. Neuronal processes of starburst amacrine cells, which express choline acetyltransferase (ChAT), form two distinct layers that can serve as anatomical landmarks (ChAT bands). Relative to these two bands, the inner plexiform layer can be divided into ten strata.^9^ Functional order exists across these strata: the dendrites of RGCs that respond to increases in visual intensity (ON cells) reside in strata 6–10, while dendrites of RGCs that respond to decreases in visual intensity (OFF cells) are found in strata 1–4. RGCs with dendrites in both divisions respond to both increases and decreases of visual intensities (ON-OFF cells).^10^ RGCs with dendrites in the outermost strata 1, 2, and 8–10 show more sustained responses to light increments or decrements, whereas those with dendrites in the innermost strata 3–7 show more transient responses.^5^^,^^6^^,^^11^ The dendrites of ON-OFF and ON direction-selective (DS) RGCs co-fasciculate with the processes of starburst amacrine cells^12^^,^^13^^,^^14^ and, therefore, co-stratify within the ChAT-positive layers 3 and 7. Dendritic co-fasciculation with starburst amacrine cell processes is a defining characteristic of these DS RGCs.^12^^,^^13^^,^^14^

Within the dLGN, retinal information was long considered to stay in “labeled lines” with each TCN receiving information from one type of RGC.^15^^,^^16^^,^^17^ Recent evidence suggests more complex and even binocular visual processing.^18^^,^^19^^,^^20^^,^^21^^,^^22^^,^^23^^,^^24^ In mice, only 28% of TCNs combine retinal inputs from one RGC type and can thus be considered as labeled lines (“relay mode”), while the remaining TCNs combine information from different RGC types, either from the contralateral eye (“combination mode”) or from both eyes (“binocular mode”).^22^

GABAergic interneurons in the dLGN differ from TCNs but also from cortical interneurons, most prominently by not only receiving synapses on their dendrites but also carrying output synapses on their dendrites. These unconventional dendritic outputs are the most prevalent output synapses of dLGN interneurons, the so-called F2 synapses.^25^^,^^26^^,^^27^ Dendro-dendritic F2 synapses are often found in a triadic arrangement, i.e., the same RGC axon synapses onto both the interneuron dendrite and the TCN dendrite, to which the interneuron provides a GABAergic inhibitory synapse in the immediate vicinity. Triadic output synapses have been found in the thalamus of many different mammalian species.^1^^,^^28^^,^^29^^,^^30^^,^^31^^,^^32^ In a three-dimensional (3D) volume of mouse dLGN containing a fully reconstructed interneuron, 94% of RGC axonal boutons that contacted the interneuron also contacted a TCN dendrite, and 90% of these retinal input synapses were found in a triadic motif, such that the interneuron provided input to the same thalamocortical dendrite as the retinal axon.^33^

In addition to having dendritic output synapses, it was suggested that the cable properties of the dLGN interneurons yield strong attenuation of signals along their dendrites.^34^^,^^35^ The abundance of dendro-dendritic output synapses, together with the predicted strong attenuation of their branched dendritic arbor, has given rise to the hypothesis that dendrites of dLGN interneurons act as “multiplexor” devices, with many independent processing units.^35^^,^^36^^,^^37^^,^^38^^,^^39^^,^^40^^,^^41^^,^^42^

Functionally, the triadic output is thought to provide fast feedforward inhibition that shapes the incoming retinal information in the time domain. This fast inhibition was suggested to provide contrast gain control,^1^ remove secondary spikes,^43^ or introduce a lag in responses that could be used to compute direction selectivity in cortex.^43^^,^^44^ The dendrites of rodent dLGN interneurons span large parts of the dLGN^33^^,^^45^ and lack the age-related pruning observed in TCNs^46^ that was suggested to contribute to the specialization of TCNs toward selected RGC types. Taken together, these properties lead to the prediction that dLGN interneurons sample retinal information broadly and unselectively along their local and multiplexing inhibitory input-output units.

In contrast to their supposed role as multiplexors, global calcium spikes as well as action potentials have been observed in dLGN interneurons *in vitro*,^47^^,^^48^^,^^49^ which points to the possibility that they might perform cell-wide signaling. We therefore asked whether dLGN interneurons indeed sample retinal information unselectively, as expected if their dendritic units are independent, or whether they exhibit a preference toward specific RGC types. Using single-cell-initiated rabies tracing from individual interneurons *in vivo*, we found that dLGN interneurons display a similar range of input specializations as TCNs.

Preference toward specific retinal inputs could result in visual response selectivity of the interneurons and contribute to a cell-wide signaling function. To test this hypothesis, we developed an approach to image the activity of dLGN interneurons *in vivo* and asked how diversely interneurons respond to visual stimuli and whether their responses are causally related to the retinal channels that provide their inputs. We found that dLGN interneurons display diverse response selectivities, and using the FRMD7 mouse mutant,^50^ which lacks horizontal direction selectivity in the retina, we show that horizontal direction selectivity of dLGN interneurons is inherited from the retina. Furthermore, response selectivities extend from the somata of interneurons into their dendrites.

Our results suggest that, instead of being multiplexors, dLGN interneurons act as selectors, receiving inputs from a defined set of RGC types that cause them to selectively represent specific visual features. Individual interneurons represent the same feature across their soma and dendrites, while different interneurons exhibit diverse features that form a continuum in the feature space. The anatomical and functional properties of feature-selective dLGN interneurons make them ideal candidates to mediate feature-selective inhibition at the first central synapse of image-forming vision.

## Results

### *In vivo* single-cell-initiated monosynaptic rabies tracing from dLGN interneurons

**Figure 1:**
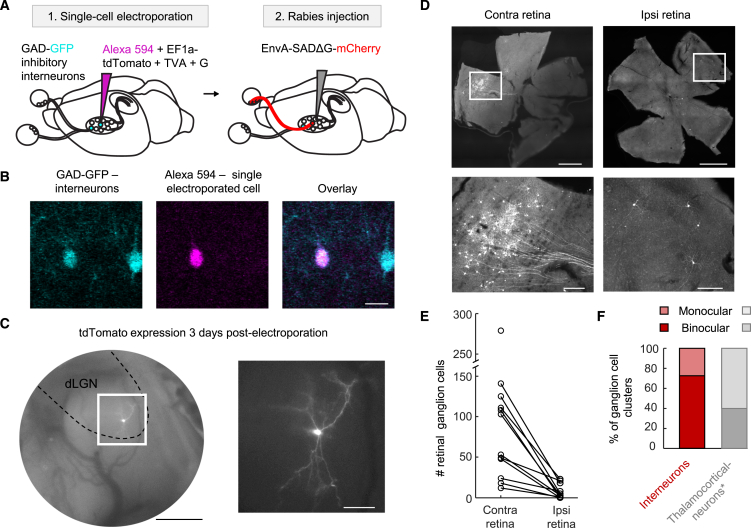
Single-cell-initiated rabies tracing from genetically identified deep-brain interneurons (A) Schematics of single-cell-initiated rabies tracing. (B) Single-cell electroporation of a GAD-positive dLGN interneuron (cyan) with Alexa 594 (magenta). Scale bar: 20 μm. (C) Electroporated interneuron displaying its characteristic morphology. Scale bars: 500 μm (left) and 100 μm (right). (D) Presynaptic RGCs in contra- and ipsilateral retinas. Top: low magnification, scale bars: 1,000 μm. Bottom: zoom in, scale bars: 200 μm. (E) Presynaptic RGC counts. (F) Percentage of monocular and binocular presynaptic RGC clusters. Data from TCNs were reported previously.^22^

To determine the number and types of RGCs that provide synaptic inputs to individual dLGN inhibitory interneurons, we developed a strategy to perform *in vivo* single-cell-initiated monosynaptic rabies tracing^51^ from genetically identified mouse dLGN interneurons. We labeled interneurons fluorescently using transgenic GAD lines—GAD65-IRES-Cre^52^ crossed to EYFP reporter (Ai3^53^) or the GAD67-GFP^54^ line—and targeted the labeled neurons by two-photon imaging for *in vivo* single-cell electroporation (Figure 1A). In each mouse, we electroporated a dLGN interneuron with fluorescent Alexa 594 dye to monitor the success of the electroporation (Figure 1B), together with three plasmids^22^^,^^51^: the avian TVA receptor, the rabies G glycoprotein, and the fluorescent protein tdTomato. Finally, we injected a G-deleted (SADΔG), EnvA-coated rabies virus expressing mCherry into the dLGN. EnvA, the ligand of TVA, permits entry of the rabies virus exclusively into the TVA-expressing cell (Figure 1C), and the G glycoprotein enables transsynaptic transfer of rabies virus to presynaptic partners of the targeted interneuron.^51^

With this approach, we mapped presynaptic RGC inputs to 12 dLGN interneurons (Figure 1D). As expected from their large dendritic size (Figure 1C; Morgan et al.^33^; Zhu et al.^45^), interneurons received inputs from a greater number of RGCs than TCNs (mean of 89.3 RGCs contralateral and 7.5 ipsilateral compared to 15.2 RGCs contralateral and 12.6 ipsilateral in TCNs; Figure 1E, TCN data from Rompani et al.^22^). Also consistent with their wide dendritic arbor, a large proportion (72.7%) of interneurons received inputs from both eyes (Figure 1F). All binocular interneurons received fewer inputs from the ipsilateral than from the contralateral retina: for all interneurons and binocular interneurons, 9.4% and 12.9%, respectively, of all presynaptic RGCs were located in the ipsilateral retina. In contrast to that observed for TCNs, the distribution of the number of RGCs in the contra- and ipsilateral retina providing input to interneurons did not deviate significantly from a random binomial distribution (Figure S1, *p* = 0.66 versus *p* = 0.004 for interneurons versus TCNs). This was consistent with the initial hypothesis that interneurons sample retinal information randomly.

**Figure 2:**
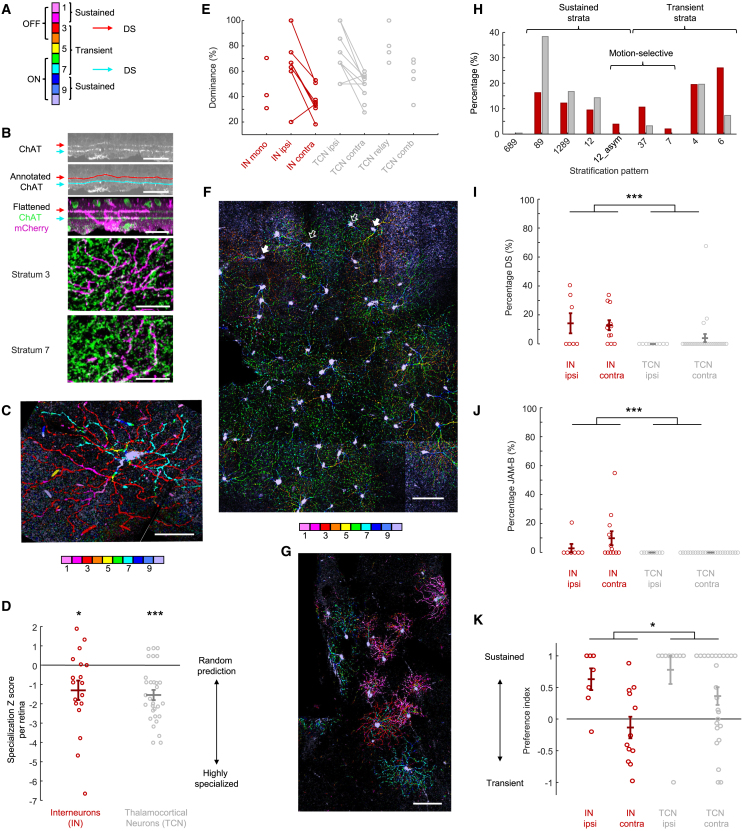
Specialization of RGC inputs to individual dLGN interneurons (A) Structure-function correlation of the inner plexiform layer strata. (B) Machine-learning-assisted detection of the ChAT bands and artificial flattening of the retina. Top to bottom: retina with ChAT bands (white); annotated ChAT bands (red and cyan); flattened z stack with ChAT bands (green) and rabies-infected ON-OFF DS RGCs (magenta); flattened z stack, interpolated at stratum 3 or 7. Scale bars: 30 μm. (C) A maximum z projection of the RGC in (B) pseudo-colored based on relative distance of the maxima from the ChAT bands. Scale bar: 30 μm. (D) Specialization *Z* scores for all retinas with RGCs presynaptic to dLGN interneurons (red) or TCNs (gray). ^∗^*p* = 0.020, ^∗∗∗^*p* < 0.001, Wilcoxon test against zero with Bonferroni-Holm correction. (E) Dominance values for interneurons (INs, red) and TCNs (gray). Values for individual retinal clusters are grouped as follows. Mono, monocular; ipsi, ipsilateral; contra, contralateral; relay, relay mode^22^; comb, combination mode.^22^ (F) Example of a contralateral RGC cluster center dominated by RGCs with dendrites in stratum 6 (green, open arrows) or 4 (orange, closed arrows). Scale bar: 100 μm. (G) Example of a contralateral RGC cluster center consisting of JAM-B (pink), ON-OFF DS (cyan and red), and ON DS (cyan) RGCs. Scale bar: 100 μm. (H) Histogram of stratification patterns of presynaptic RGCs transsynaptically labeled from INs (red) or TCNs (gray). Stratification “xyz” refers to RGCs stratifying in retinal strata x, y, and z. (I and J) The percentage of DS (I) and JAM-B (J) inputs to INs (red) and TCNs (gray) from ipsi- and contralateral retinas. ^∗∗∗^*p* < 0.001, Mann-Whitney U test. (K) Sustained over transient preference indices for RGC clusters in ipsi- and contralateral retinas labeled from INs (red) and TCNs (gray). ^∗^*p* = 0.039, Mann-Whitney U test. (D, E, H, I, J, and K) *n* = 638 RGCs presynaptic to 12 dLGN interneurons; *n* = 245 RGCs presynaptic to 15 TCNs. Horizontal lines: group averages, error bars: ± SEM. TCN data from Rompani et al.^22^

To understand which RGC types provide inputs to dLGN interneurons, we classified the presynaptic RGCs based on the stratification of their dendrites within the inner plexiform layer (Figure 2A). We took high-resolution confocal stacks of the presynaptic RGCs in flat-mounted retinas co-stained with antibodies against mCherry and ChAT (Figure 2B). Previous analysis of the rabies-traced RGCs^22^ had two bottlenecks. First, the assignment of dendrites to individual cells required manual segmentation. Second, the stratification of dendrites within the inner plexiform layer was assessed based on successive side projections, which were also performed manually, of the segmented dendrites. Both bottlenecks arise from the 3D nature of the flat-mounted retinas, in which the ChAT bands are curved planes (Figure 2B). To overcome these limitations, we trained a convolutional neural network, U-Net,^55^ to automatically detect the ChAT bands. Knowing the ChAT-band locations in the z axis, we could virtually flatten the image stacks (Figure 2B). The dendrites of mCherry-labeled RGCs could then, in a single top-down maximum projection, be automatically labeled with different colors according to their depths relative to the ChAT bands (Figure 2C). In this pseudo-colored top-down projection, dendrites belonging to a given RGC can be recognized by their emergence from the soma in a snowflake-crystal pattern, and the dendrites can be followed traveling through the layers of the inner plexiform layer as their color changes in a rainbow sequence (Figures 2C and S2). For binocular cells, we determined the RGC types separately in both eyes, and we refer to the presynaptic RGCs in each retina as “cluster” since the RGCs were typically centered in one quadrant (Figure 1D). We classified 638 RGCs labeled in single-cell-initiated rabies tracings from dLGN interneurons.

### Specialization of RGC inputs to dLGN interneurons

We used two approaches to quantify the specialization of RGC inputs to dLGN interneurons. First, we measured the difference in the number of RGC types presynaptic to each interneuron from that expected by a random draw. For each number of presynaptic RGCs, we simulated a random draw from the empirically found overall distribution of RGC types using Monte Carlo simulation. The number of RGC types expected from a random draw depends on the total number of RGCs (Figure S2). The deviation of the empirically found number of RGC types to the number of RGC types expected by a random draw was quantified by a specialization *Z* score (measured minus expected number, divided by the simulated standard deviation [SD]). A *Z* score of zero means that the inputs match a random prediction, and a negative *Z* score indicates that the inputs are specialized toward certain RGC types. Given the multiplexor model, we expected the dLGN interneurons to randomly sample RGC types and, therefore, to have *Z* scores around zero. In contrast, we found the *Z* scores of interneurons and TCNs to be similarly distributed—with some more randomly sampling cells around zero and several highly specialized cells and retinal clusters with *Z* scores smaller than −2 (Figures 2D and S2; median *Z* scores significantly below zero; Wilcoxon test, interneurons: *p* = 0.022 and TCNs: *p* < 0.001).

Second, we analyzed interneuron input specialization by quantifying RGC-type dominance. Within a presynaptic cluster of RGCs, RGC types can be present in similar numbers, or the cluster can be dominated by few RGC types. We defined dominance as the relative abundance of the most prevalent RGC type, such that clusters dominated by individual RGC types would have dominance values close to 100%, whereas clusters with equal representation of cell types would have lower dominance (e.g., 20% for five cell types). Note that dominance does not take into account the relative strengths of the different cell types that provide input. In analogy to TCNs, in which we had found monocular relay-mode and combination-mode clusters, as well as ipsi- and contralateral binocular clusters (“TCN relay,” “TCN comb,” “TCN ipsi,” and “TCN contra,” respectively),^22^ we differentiated here for interneurons between monocular, binocular ipsilateral, and binocular contralateral clusters (“IN mono,” “IN ipsi,” and “IN contra,” respectively). The dominance of presynaptic RGC clusters was quantitatively comparable between interneurons and TCNs (Figure 2E); similar dominance values were found in interneurons and TCNs for both ipsilateral clusters (mean 65% and 73%, Mann-Whitney U test, *p* = 0.49) and contralateral clusters (mean 37% and 48%, Mann-Whitney U test, *p* = 0.083). Also, monocular presynaptic clusters of interneurons (IN mono) and monocular combination-mode clusters of TCNs (TCN comb) exhibited similar dominance values (mean 48% and 52%, Mann-Whitney U test, *p* = 0.88), consistent with the fact that we observed no interneurons receiving relay-mode inputs formed by only one RGC type. Even highly specialized interneurons received inputs from more than one RGC type (Figures 2F and 2G). Most (86%) binocular interneurons showed higher dominance values for ipsilateral than contralateral clusters, similar to those observed for binocular TCNs (78%, mean difference contra-ipsi 28% [IN] and 24% [TCN], Mann-Whitney U test, *p* = 0.61).

While the degrees of specialization between interneurons and TCNs were similar, the sources of specialization were complementary (Figure 2H). We anatomically classified the RGCs based on their stratification as putatively motion selective by the following rules: we defined RGCs co-fasciculating with ChAT-positive processes as DS (ON-OFF DS RGCs, type 37, and ON DS RGCs, type 7). We defined RGCs stratifying in strata 1 and/or 2 and having asymmetric dendritic arbors as JAM-B cells, which were described previously as orientation-selective (OS) or DS cells.^56^^,^^57^

Interneurons received more ON-OFF DS, ON DS, and JAM-B RGC inputs than TCNs (Figure S2). The percentage of these inputs was 15.2% for interneurons (8.8% ON-OFF DS, 2.2% ON DS, and 4.2% JAM-B) as opposed to 3.3% for TCNs (3.3% ON-OFF DS, Fisher’s exact test, *p* < 0.001). The percentages of DS inputs (ON-OFF DS or ON DS) per retina were significantly higher for interneurons than for TCNs (Mann-Whitney U test, *p* < 0.001, Figure 2I), as well as the percentage of JAM-B inputs (Mann Whitney U test, *p* < 0.001, Figure 2J). The percentage of interneurons receiving at least one DS or JAM-B RGC input (83%) was also significantly higher than for TCNs (Figure S2, 12%, Fisher’s exact test, *p* < 0.001). For 7 of 12 experiments, we performed post hoc immunostaining with Cart antibody (Figure S1), which labels a subset of DS RGCs.^58^^,^^59^ In these retinas, 10.6% (43/404) of presynaptic RGCs were morphologically ON-OFF DS (compared to 10.8% among all retinas) and 7.9% (54/687) of all; 6.9% (28/404) of all morphologically classified presynaptic RGCs were Cart positive. In total, 85.7% (24/28) of Cart-positive RGCs were bistratified, including 75% (18/24) of ON-OFF DS cells co-fasciculating with ChAT-positive processes in strata 3 and 7 (Figure 2B) and 25% (6/24) other bistratified cells. Therefore, 64.3% (18/28) of all Cart-positive RGCs presynaptic to dLGN interneurons were ON-OFF DS cells. Of all ON-OFF DS cells, 41.9% (18/43) were Cart positive (Figure S1), indicating that this population contained different subtypes of ON-OFF DS cells.^59^^,^^60^^,^^61^^,^^62^ The remaining Cart-negative ON-OFF DS cells (Figure S1) accounted for 6.6% (25/376) of all Cart-negative RGCs. These results indicate that dLGN interneurons receive a substantial fraction of inputs from diverse motion-selective RGC subtypes.

We then anatomically classified RGCs based on their stratification as putatively transient or sustained by the following rule: RGCs with dendrites in the innermost strata 3–7 were defined as transient, while those in the outermost strata 1, 2, and 8–10 as sustained.^5^^,^^6^^,^^11^^,^^63^^,^^64^ Interneurons received fewer sustained and more transient RGC inputs than TCNs: 58% of retinal inputs to interneurons, compared to 30% to TCNs, were transient (Figure S2, Fisher’s exact test, *p* < 0.001). The interneuron’s preference for transient inputs was especially pronounced in the contralateral inputs (62%). RGC inputs from the ipsilateral retina, on the other hand, showed a preference toward sustained inputs, similar to the ipsilateral inputs to TCNs, albeit with a slightly higher relative contribution of transient inputs (13% versus 3.6%). We calculated preference indices for individual retinas (Figure 2K) defined as the difference between RGC numbers divided by their sum: (# sustained − # transient)/(# sustained + # transient). A preference index of 1 indicates exclusively sustained, and a preference index of −1 indicates exclusively transient RGCs in a cluster. Preference indices spread widely, but the majority (58%) of contralateral retinas showed a preference for transient information, while 86% of ipsilateral retinas had a preference for sustained information and 43% even received exclusively sustained information (preference index = +1). The preference indices for retinal clusters presynaptic to interneurons were significantly shifted toward transient information compared to that of TCNs (Mann-Whitney U test, *p* = 0.039, Figures 2K and S2).

Analogously, we defined an ON-OFF preference index as a weighted sum of inputs divided by the total input number: (# ON × 1 + # OFF × 0 + # ON-OFF × 0.5)/(# ON + # OFF + # ON-OFF). Individual interneurons showed different degrees of preference for ON or OFF inputs. On average, contralateral inputs to interneurons had no ON-OFF preference (index mean ± SD: 0.51 ± 0.14), similar to inputs to TCNs (Figure S2). Ipsilateral inputs to interneurons displayed an ON preference (index mean ± SD: 0.79 ± 0.14).

To predict the influence that ipsilateral inputs could have on the overall specialization of interneurons, we calculated the preference indices of all inputs per interneuron and compared them to preference indices calculated for contralateral inputs only (Figure S2). The change in preference indices introduced by ipsilateral inputs was small (mean absolute change: 0.05 for transient/sustained, 0.03 for ON/OFF, assuming equal contribution, which could overestimate their influence^65^), suggesting that input specialization to dLGN interneurons is mostly determined by the diversity of contralateral presynaptic RGCs.

Finally, to correlate the variety of morphologically defined RGC types providing input to dLGN interneurons (Figure 2H) with molecularly defined RGC types, we performed rabies tracing simultaneously from several interneurons. We injected into the dLGN of GAD65-IRES-Cre mice conditional adeno-associated viruses (AAVs) expressing TVA and G glycoprotein,^66^ followed by EnvA-SADΔG-mCherry rabies virus, and subsequently performed immunohistochemistry with Cart,^58^ Satb1,^61^^,^^67^ Satb2,^60^^,^^61^ and SMI-32^68^ antibodies in the retina (Figure S1). In addition, GABAergic RGCs^69^ were labeled genetically in a subset of experiments. 29.9% of presynaptic RGCs were positive for the alpha RGC marker SMI-32^68^^,^^70^^,^^71^ (504/1687, *n* = 4), the majority of which were of morphological type 4 (76%, 19 of 25). For comparison, we had found 19.5% of morphological type 4 presynaptic RGCs in the single-cell-initiated rabies tracing. Using markers for ON-OFF DS RGCs, we found that 11.1% of presynaptic RGCs were Satb1 positive (395/3560, *n* = 8), 12.9% were Satb2 positive (68/528, *n* = 5), and 12.5% were Cart positive (106/851, *n* = 7). For comparison, we had found 10.8% of morphological type 37—with starburst amacrine cells co-fasciculating—RGCs and 7.9% Cart-positive RGCs providing inputs to individual dLGN interneurons by single-cell-initiated rabies tracing. We morphologically classified Cart-positive RGCs presynaptic to dLGN interneurons and found that 92.0% (23/25) were bistratified, including 60.0% (15/25) with morphological type 37, consistent with Cart labeling ON-OFF DS but also additional RGC types among those that provide input to dLGN interneurons. For comparison, we had found 64.3% (18/28) morphological type 37 among Cart-positive RGCs by single-cell-initiated rabies tracing. Of all ON-OFF DS cells from an unbiased selection of RGCs presynaptic to dLGN interneurons (agnostic of their Cart label), 35.0% (7/20) were Cart positive, as compared to 41.9% (18/43) in the single-cell-initiated rabies tracing, confirming that different subtypes of ON-OFF DS cells^59^^,^^60^^,^^61^^,^^62^ provide input to dLGN interneurons. In contrast to all other markers, only 1.6% of presynaptic RGCs were GABAergic (57/3,512, *n* = 6). In addition, we performed anterograde synaptic tracing from Cart-positive RGCs by injecting AAV-flex-mWGA-mCherry intravitreally^72^ into Cart-IRES2-Cre-D mice. We found mCherry signal in a subset of dLGN interneurons (Figure S1), confirming that dLGN interneurons receive input from Cart-positive RGCs.

Taken together, dLGN interneurons displayed a wide range of input specializations. Although the degrees of specialization resembled those of TCNs, they displayed an overrepresentation of transient information and a significantly larger proportion of motion-selective inputs.

### dLGN interneurons are functionally specialized *in vivo*

**Figure 3:**
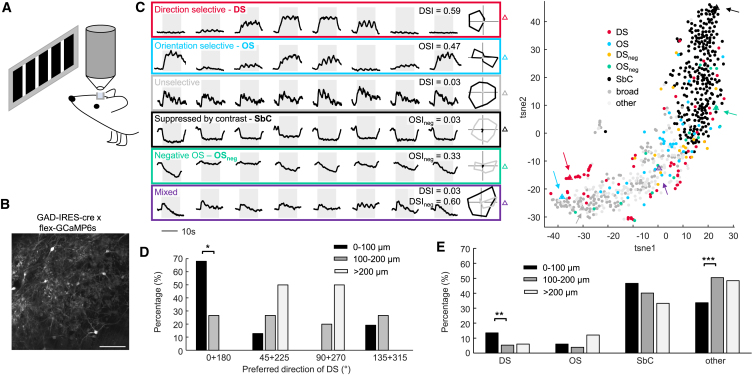
*In vivo*, dLGN interneurons are specialized toward diverse visual features (A) Schematics of *in vivo* calcium imaging in the dLGN. (B) Example field of view with GCaMP6s-expressing dLGN interneurons. Scale bar: 100 μm. (C) Responses of 6 example interneuron somata (left panel) to gratings drifting in 8 different directions with 400 μm/s during the shaded 12 s interval. Polar plots indicate the positive (black) and negative (gray) response amplitudes for the respective motion direction. Two-dimensional (2D) t-distributed stochastic neighbor embedding (t-SNE) plot of interneuron response vectors (right). Triangles with arrows indicate the response vectors of the 6 example interneurons shown left in matching colors. (D) Histogram of the preferred directions of DS interneurons at three different imaging depths (*n* = 47, 15, 2 for 0–100, 100–200, >200 μm). ^∗^*p* = 0.02, Fisher’s exact test for horizontal (group 1 of 4) against all other directions (groups 2–4 pooled), compared between any two depths, Bonferroni-Holm corrected. (E) Histogram of response categories: DS, OS, SbC, and all others, at three different imaging depths (*n* = 347, 281, 33 for 0–100, 100–200, >200 μm). ^∗∗^*p* = 0.007, ^∗∗∗^*p* < 0.001, Fisher’s exact test for each category against all others (for example, DS versus non-DS), compared between two depths, Bonferroni-Holm corrected. (C, D, and E) Interneurons recorded in 14 wild-type mice under anesthesia.

The diverse specialization of the RGC types providing input to individual interneurons could result in diverse functional specializations. To test this, we established *in vivo* recordings from mouse interneurons by applying two-photon calcium imaging in a mouse line in which GCaMP6s expression was limited to interneurons (GAD65-IRES-Cre^38^) crossed with Ai94D^53^ and CAG-stop-tTA2.^73^ We then implanted a glass cylinder, closed at the inner end with a glass coverslip, on top of the dLGN (Figure 3A). This preparation allowed visualization of GCaMP6s-expressing neurons in a field of view of 450 × 450 μm (Figure 3B). We recorded visually evoked responses from dLGN interneuron somata (Figure 3C) to a set of stimuli consisting of black-and-white gratings drifting in 8 different directions at 3 different velocities (400, 1,200, and 2,400 μm/s on the retina).

We observed a wide range of different visual responses to the 24 different stimuli. Some interneurons were unselective for the presented stimuli while others were DS, OS, or suppressed by contrast (SbC) (Figure 3C). Yet other interneurons had mixed responses such as positive-and-negative or phasic-and-tonic responses to the different stimuli, which could result in SbC responses with orientation or direction selectivity of the negative amplitudes (Figure 3C). We quantified orientation or direction selectivity of negative amplitudes analogously to positive amplitudes and refer to the selectivity indices as positive direction selectivity index (DSI), negative direction selectivity index (DSI_neg_), positive orientation selectivity index (OSI), or negative orientation selectivity index (OSI_neg_). We additionally calculated a significance metric (*p* value) for all four selectivity indices (denoted pDS, pOS, pDS_neg_, and pOS_neg_) by randomly permuting individual trials across stimulus orientations, and we considered only interneurons for which the index was larger than 95% of the shuffled control data (*p* < 0.05, Figure S3) as selective. For assigning a unique category for each interneuron (DS, OS, DS_neg_, OS_neg_, SbC, and broad), we introduced a hierarchy based on DSI, OSI, DSI_neg_, OSI_neg_, and %SbC from high to low, such that a cell would be assigned to the highest category for which it met the criteria. We then performed dimensionality reduction of the 48-dimensional response vectors defined by the positive and negative response amplitudes to the 24 stimuli (example in Figure S3). We found no clear separation of the response vectors into clusters; rather, they formed a continuum distributed across a wide feature space (Figures 3C and S3). The SbC feature exhibited a bimodal distribution, but the two distributions overlapped, and interneuron examples could be found with intermediate percentages of suppressed responses. 9% and 11% of SbC interneurons within the upper 100 μm had strong selectivity for direction or orientation (Figure S3). Altogether, the functional properties of interneurons *in vivo* were diverse, with different features merging into each other.

Previous studies have indicated a dorsoventral gradient from superficial to deeper dLGN for DS RGC projections,^56^^,^^58^^,^^74^^,^^75^^,^^76^^,^^77^ as well as an overrepresentation of DS and/or OS responses in the superficial dLGN,^78^ in particular an overrepresentation of horizontal motion in the superficial dLGN.^79^ Therefore, we asked whether dLGN interneuron specialization is also dependent on depth. Superficial DS interneurons (0–100 μm depth) displayed an overrepresentation of horizontal motion (68% preferring 0° or 180° ± 22.5°, Figure 3D). Deeper DS interneurons (100–200 μm depth), on the other hand, showed a significant shift (chi-squared test, *p* = 0.002) away from horizontal motion (Figure 3D) (26.7% preferring 0° or 180° ± 22.5°; Fisher’s exact test with Bonferroni-Holm correction, *p* = 0.02). The distribution of the four categories—DS, OS, SbC, and others—also changed for deeper relative to superficial interneurons (chi-squared test, *p* < 0.001), with a significant drop in the proportion of significantly DS neurons (from 13.5% at 0–100 μm to 5.3% at 100–200 μm, Fisher’s exact test, *p* = 0.007 after Bonferroni-Holm correction) (Figure 3E). To avoid artifacts due to different recording depths, we always refer to the data recorded within the upper 100 μm when comparing between groups.

**Figure 4:**
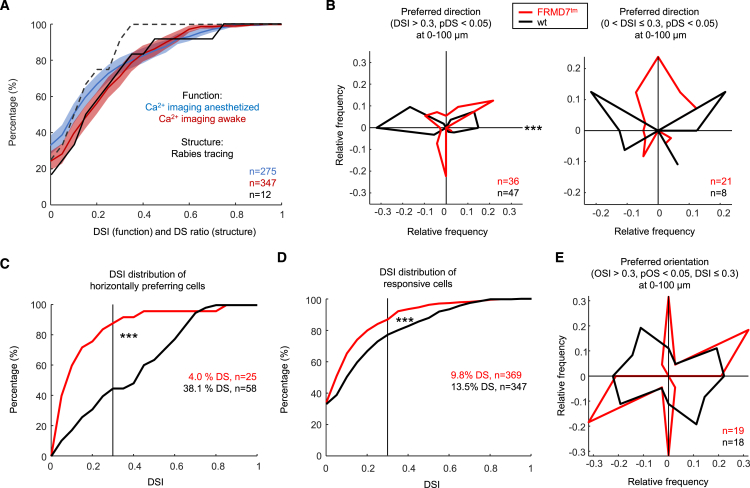
Horizontal direction selectivity of dLGN interneurons is mostly inherited from the retina (A) Cumulative histograms of the ratio of DS cells (DS ratio) in individual presynaptic retinal clusters from rabies tracing (black) and DSI values from calcium imaging in awake (red) and anesthetized (blue) animals. Full black line: DS ratio including JAM-B, dotted black line: excluding JAM-B. Shaded area: mean ± SD. The y offset indicates the percentage of cells with no positive response with SNR > 2.5 (imaging data) or no DS inputs (rabies tracing). *n* = 275 interneurons for calcium imaging in anesthetized, *n* = 347 for imaging in awake animals. *n* = 638 RGCs presynaptic to *n* = 12 interneurons for rabies tracing. (B) Left: polar plot of preferred directions of DS interneurons (DSI > 0.3, pDS < 0.05) in wild-type (WT, black) and FRMD7™ (red) mice. Right: polar plot of the preferred directions of interneurons with significant directional bias (0 < DSI ≤ 0.3, pDS < 0.05) in wild-type (WT, black) and FRMD7™ (red) mice. ^∗∗∗^*p* < 0.001, Fisher’s exact test, horizontal directions against all others. (C) Cumulative histogram of DSI for all horizontal axis-preferring (0° or 180° ± 15°) interneurons with SNR > 2.5 of the positive response in wild-type (WT, black) and FRMD7™ (red) mice. ^∗∗∗^*p* < 0.001, Kolmogorov-Smirnov test. (D) Cumulative histogram of DSI in wild-type (WT, black) and FRMD7™ (red) mice. ^∗∗∗^*p* < 0.001, Kolmogorov-Smirnov test. DSI was set to zero if the SNR of the positive response was ≤2.5. (E) Polar plot of preferred orientations of OS, not DS interneurons (OSI > 0.3, pOS < 0.05, DSI ≤ 0.3) in wild-type (WT, black) and FRMD7™ (red) mice. Fisher’s exact test, horizontal directions against all others, n.s., chi-squared test, n.s. (A–E) Interneurons, number annotated as n, recorded at 0–100 μm depth in 14 wild-type and 5 FRMD™ mice; (B–E) under anesthesia.

In addition to anesthetized recordings, we also recorded visual responses in awake mice. We found dLGN interneurons of all categories in awake mice, with slightly altered distribution of categories (chi-squared test, *p* = 0.002). There was a small reduction in the percentage of SbC interneurons from 42.7% to 35.6% (Fisher’s exact test, non-significant [n.s.]), the opposite effect of what has been observed for thalamocortical dLGN neurons.^80^ This change was accompanied by more interneurons displaying positive responses (76% instead of 67% with signal-to-noise ratio [SNR] > 2.5 of the positive amplitudes, Fisher’s exact test, *p* = 0.02) and fewer interneurons displaying negative responses (73% instead of 80% with SNR > 2.5 of the negative amplitudes, Fisher’s exact test, *p* = 0.055), resulting in an overall right-shift (Figure 4A) (Mann-Whitney U test, *p* = 0.02) in the distribution of DSI values (which were by definition zero for interneurons with no positive response having SNR > 2.5). Velocity preference and the distribution of response amplitudes were similar under awake and anesthetized conditions (Figure S3) (chi-squared test, n.s., and Kolmogorov-Smirnov test, n.s.), but there were fewer cells with high SNRs in awake (Figure S3, Kolmogorov-Smirnov test, *p* = 0.02).

To understand how the functional specialization of individual interneurons is related to the RGC types providing their retinal inputs, we first compared the distribution of the percentage of DS inputs to individual interneurons, as revealed by monosynaptic rabies tracing, to the distribution of the direction selectivity indices of individual interneurons, determined using functional imaging. 13.5% of interneurons in the superficial 100 μm of the dLGN were strongly DS (DSI > 0.3, pDS < 0.05) in anesthetized animals, and 9.1% of superficial interneurons were strongly DS in awake animals (Fisher’s exact test, n.s.). Remarkably, the distribution of DS indices matched the distribution of the percentage of DS inputs received by individual interneurons (Figure 4A). This structure-function correlation indicates that somatic responses reflect the overall distribution of dendritic inputs.

### Anatomical and functional specialization of dLGN interneurons are causally related

To determine whether the relationship between RGC input specialization and interneuron functional specialization is causal, we used FRMD7™ mice in which horizontal direction selectivity is absent in the retina but vertical direction selectivity remains intact.^50^ DS interneurons (DSI > 0.3, pDS < 0.05) of FRMD7™ mice showed a significant change in the distribution of preferred directions compared to wild-type mice (chi-squared test, *p* < 0.001) (Figures 4B and S4), with a strong reduction in the percentage of DS interneurons preferring horizontal (0° ± 15° and 180° ± 15°) motion directions (from 46.8% to 2.8% at 0–100 μm depth, Fisher’s exact test, *p* < 0.001). Also the directional bias of significantly but less well-tuned (0 < DSI ≤ 0.3, pDS < 0.05) interneurons was reduced in the horizontal direction (Figure 4B) (from 25% to 5%, Fisher’s exact test, n.s.), and all interneurons preferring to any degree horizontal motion were less sharply tuned to horizontal motion (Kolmogorov-Smirnov test, *p* < 0.001) (Figures 4C and S4) with a significant reduction of cells with significant direction selectivity (from 38.1% to 4.0%, Fisher’s exact test, *p* = 0.001). This resulted in an overall decrease in the percentage of DS neurons (from 13.5% in wild-type to 9.8% in FRMD7™ mice, Kolmogorov-Smirnov test, *p* < 0.001) (Figures 4D and S4). Interneurons that were OS, but not DS, did not display a reduction in horizontal preference in FRMD7™ (Figure 4E). Mice heterozygous for the mutant allele displayed an intermediate phenotype (Figure S4). Taken together, the experiments with FRMD7™ mice suggest, at least for direction selectivity, that RGC input specialization and interneuron functional specialization are causally linked.

### dLGN interneurons display visual features globally

**Figure 5:**
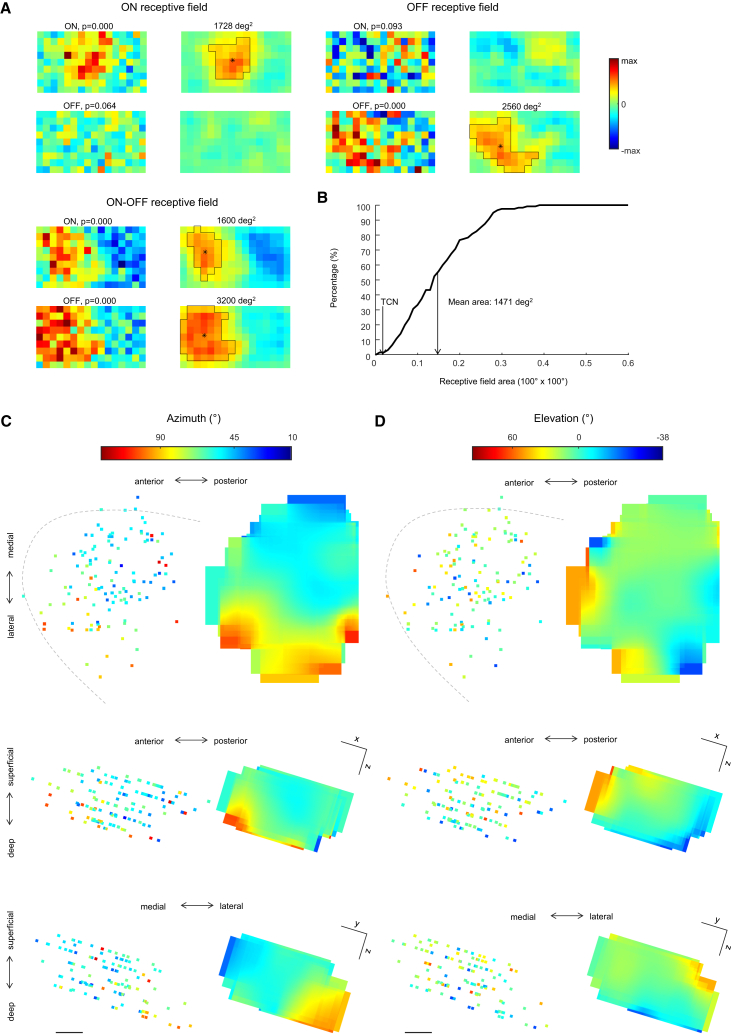
dLGN interneurons have large receptive fields and are retinotopically arranged (A) Receptive fields of 3 interneuron somata in the dLGN. Left: response amplitude (positive if |maximum| ≥ |minimum|, negative if |minimum| > |maximum|), normalized to the maximum absolute ON or OFF response (denoted “max” in the scale bar); *p* values denote the significance of the local correlations with respect to a shuffling control; pixel size 8° × 8°. Right: left panels with 3 × 3 average filters. Black outlines: connected region around the maximum with pixel values above half maximum (receptive field); stars: receptive field center of mass. (B) Cumulative histogram of the receptive field sizes of all recorded interneurons with significant (*p* < 0.05) ON or OFF receptive field. Arrow labeled TCN annotates the average receptive field size of TCNs.^81^ (C and D) Retinotopic arrangement of the recorded dLGN interneurons in horizontal planes (upper) and across depth (lower, rotated to match the angle of window implantation). Azimuth (C) and elevation (D) refer to the center of mass of the receptive field. Left: individual interneurons with overlapping data averaged. Right: interpolated retinotopic maps. Scale bars: 100 μm. (A–D) Interneurons recorded in 4 wild-type mice under anesthesia.

We mapped the receptive fields of dLGN interneurons with sparse noise. As expected from the high convergence (Figure 1E), their receptive fields were large in comparison to TCNs^78^^,^^81^^,^^82^^,^^83^ (Figures 5A and 5B), covering 1,471 ± 823 degrees^2^ (mean ± SD) of visual space. The receptive field center positions correlated with the positions of interneuron somata (Figure 5C), broadly matching the known retinotopic map in the dLGN.^78^

**Figure 6:**
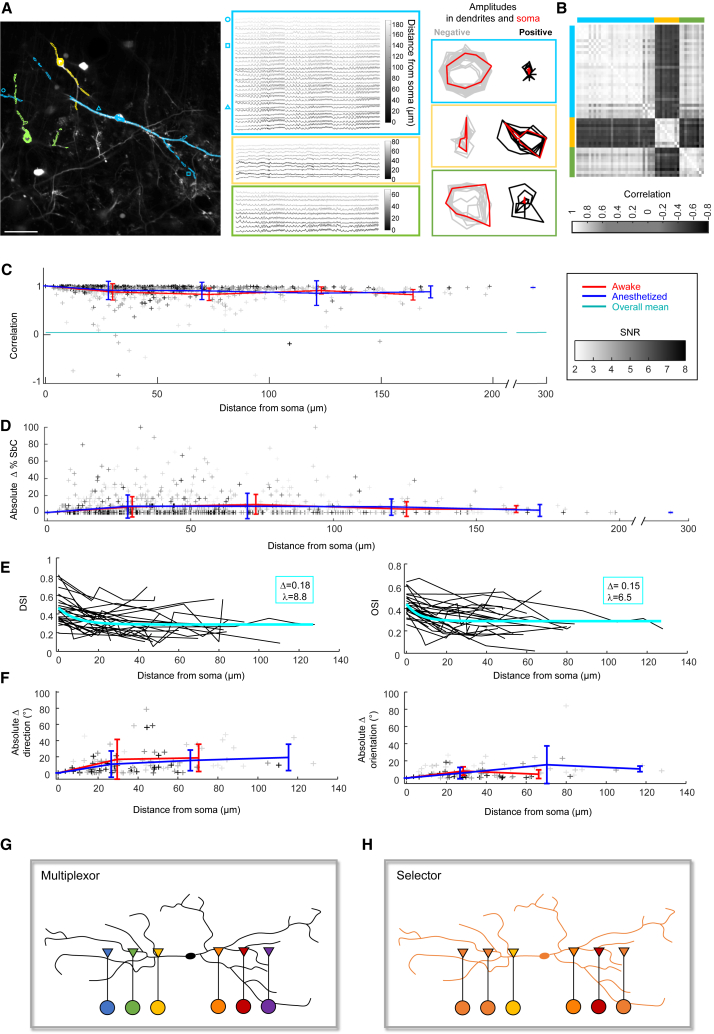
Somatic visual features extend into the dendrites (A) Left: 3 example interneurons with their annotated dendritic regions (awake recording). Scale bar: 50 μm. Middle: calcium responses to visual stimulation recorded at different dendritic regions. Frame color: interneuron identity on the left. Gray scale: Euclidean distance from soma. Symbols: selected positions on the left. Right: polar plot of positive (right, black) and negative (left, gray) visual response amplitudes at different dendritic regions to gratings drifting at 400 μm/s velocity in 8 different directions. Red: somatic response polar plots. (B) Correlation matrix for the 16-dimensional vectors of positive and negative response amplitudes of the 3 example interneurons in response to gratings drifting at 400 μm/s velocity in 8 different directions. Colors: interneuron identity from (A). (C) Response correlation of all annotated dendritic compartments with their respective soma (16-dimensional response vector), plotted against Euclidean distance from the soma. Gray scale: SNR. Red/blue error bars: mean ± SD under awake (red) or anesthetized (blue) conditions in 50 μm bins. Cyan line: mean of the full correlation matrix of all 1,135 compartments. For each interneuron, the speed which evoked the largest absolute response was selected. (D) Absolute differences in the percentage of SbC responses in dendritic compartments with respect to soma, plotted against Euclidean distance from soma. Gray scale: SNR. Red/blue error bars: mean ± SD under awake (red) or anesthetized (blue) conditions in 50 μm bins. (E) DSI (left) and OSI (right) values of interneuron dendritic compartments, plotted against Euclidean distance from the soma. Cyan: mono-exponential fit with length constant λ and amplitude Δ as indicated (y = Δ⋅exp(−x/λ) + y_0_). (F) Absolute differences in preferred direction (left) or preferred orientation (right) between dendritic compartments and soma, plotted against Euclidean distance from the soma. Red/blue error bars: mean ± SD under awake (red) or anesthetized (blue) conditions in 50 μm bins. (E and F) Included are cells with any compartment showing DSI > 0.3 (left) or OSI > 0.3 (right). For each interneuron, the speed that evoked the highest DSI (left) or OSI (right) at the soma was selected. (G) Multiplexor model, with many independent and functionally distinct units along the dendrites. Triangles symbolize the triadic units consisting of RGC inputs to both the interneuron dendrite and the TCN (not shown), which in turn receives dendro-dendritic inhibitory synapses from the interneuron. (H) Selector model, in which the interneuron receives functionally specialized inputs from the retina, which dictate its cell-wide functional specialization. (C–F) Data from *n* = 227 interneurons with 1,339 compartments recorded in 5 wild-type, 5 hemizygous FRMD7™, and 3 heterozygous FRMD7™ mice, of which 222 interneurons and 1,135 compartments with SNR > 2.5 were included.

Considering what the function of the strong specializations we observed at interneuron somata might be, it is an intriguing possibility that they may influence local feedforward inhibition at the dendro-dendritic output synapses. A necessary requirement for this would be that the somatic specializations are also present in the dendrites. *In vitro*, it has indeed been shown that dLGN interneurons exhibit backpropagating dendritic spikes.^47^ To test whether the somatic specializations also extend into the dendrites *in vivo*, we annotated the dendrites that ran parallel to the imaging plane for individual interneurons (Figure 6A). We annotated 1,339 dendritic compartments from 227 interneurons of which 1,135 fulfilled the SNR > 2.5 criterion. Somatic and dendritic responses were similar (Figure 6A), as reflected by higher correlations within than between interneurons (Figure 6B). Correlations dropped slightly from 0.94 to 0.86 between 1 and 75 μm from the soma (Spearman r^2^ < 0.04, Figure S5) but did not significantly decrease further along the dendrites beyond 75 μm distance (Spearman r^2^ < 0.005, *p* > 0.3). Average correlations of the response vectors remained higher than 0.8 along the dendrites, in both anesthetized and awake animals (Figure 6C). The percentage of SbC responses was stable with average deviations equal or less than 5.4% and average absolute deviations equal or less than 15% (Figures 6D and S5). The DS and OS indices of neurons with DSI > 0.3 or OSI > 0.3 decreased slightly within the first 20 μm but then stabilized along the dendrites (Figures 6E and S5). The preferred directions/orientations of the responses were stable with average absolute deviations of less than 25° along the dendrites (Figures 6F and S5). Therefore, dLGN interneurons are functionally specialized not only at their soma but also at their dendrites, and the two specializations match.

## Discussion

We performed single-cell-initiated rabies tracing from and two-photon imaging of dLGN inhibitory interneurons *in vivo* in order to understand how they integrate retinal inputs, both anatomically and functionally. We found that individual interneurons are anatomically specialized at a similar level as TCNs and are functionally specialized to encode diverse visual features, forming a continuum of responses to the same stimuli.

### “Multiplexor” versus “selector” interneurons

Due to the triadic output arrangements and predicted electrotonic attenuation of their dendrites, interneurons in the dLGN have been thought to act as multiplexors.^35^^,^^36^^,^^37^^,^^38^^,^^39^^,^^40^^,^^41^^,^^42^ In such a multiplexor model (Figure 6G), the dendritic triads across the dendritic tree of interneurons act as independent units, receiving input from and shaping the thalamocortical outputs belonging to a (presumably random) multitude of retinal channels. However, our anatomical and functional results are most consistent with dLGN interneurons being selectors. In a selector model (Figure 6H), the interneuron receives inputs from a defined subset of retinal channels, which causes the interneuron to respond selectively to the visual feature determined by the given subset of retinal channels. This can be a small subset of retinal channels resulting in a feature of narrow selectivity or a larger subset containing a diverse subsample of retinal channels compatible with a random draw and presumably resulting in a feature of broad selectivity. The visual feature arising in the soma of a selector interneuron may backpropagate into the interneuron dendrites and result in a cell-wide functional specialization. The selector model is supported by the following findings. First, the number of RGC types providing input to most dLGN interneurons was smaller than expected by a random draw. Retinal inputs to interneurons displayed a similar degree of specialization as retinal inputs to TCNs, reflected by the distribution of specialization scores for RGC types (Figure 2D), cell-type dominances (Figure 2E), and preference indices for transient versus sustained inputs (Figure 2K). As an example, we found interneurons that were highly specialized for receiving input from motion-selective RGCs (Figures 2G, 2I, and 2J). Second, dLGN interneurons displayed a wide variety of response selectivities *in vivo*, including highly specialized responses such as DS or OS responses (Figure 3). Third, horizontal direction selectivity of dLGN interneurons was strongly reduced in mice lacking horizontal direction selectivity in the retina (Figures 4B and 4C), confirming a causal relationship between presynaptic retinal specialization and the postsynaptic visual feature. Fourth, the selectivities of dendrites of individual dLGN interneurons and their somata were similar (Figures 6A–6F), consistent with a cell-wide functional specialization.

The multiplexor model combines two assumptions. The first assumption is that interneurons receive a diverse multitude of retinal inputs, and the second is that different triadic units are mutually independent. The first assumption is violated for the subset of dLGN interneurons displaying high input specialization, while it is still compatible with those interneurons displaying more random sets of inputs and visual features of low selectivity. The second assumption is potentially violated by all dLGN interneurons, since our data show that the somatic visual features extend far into the dendrites (Figures 6A–6F)—consistent with the existence of backpropagating spikes,^47^ which could shape or even initiate^49^ dendritic inhibition provided by the interneurons. It remains to be shown to what degree individual triadic elements and distal dendritic compartments could still be electrically isolated from the global dendritic signals in the incoming and/or outgoing direction and could thereby reflect remaining variability from within the subset of selected retinal channels that they receive (e.g., differences between ON-OFF DS, ON DS, and JAM-B inputs in Figure 2G). Due to delays for dendritic conduction and/or integration, it is likely that the feature-selective inhibition triggered by cell-wide features would act at the slower timescales of non-locked^43^ or sustained^49^ inhibition provided by the dLGN interneurons, possibly leaving short time windows for independent multiplexing of diverse inputs even in selector interneurons.

### Inheritance versus *de novo* computations

Selection of retinal input channels can result in a visual feature in dLGN interneurons that is similar to the dominant retinal input channels but can also result in the computation of a novel visual feature. Evidence for *de novo* computation comes from the combined positive/negative and tonic/phasic responses in individual interneurons (Figure 3C).

While *de novo* computations may reflect computations based on retinal inputs, *de novo* computation may also require extraretinal inputs, such as lateral inhibition between dLGN interneurons, excitation by thalamocortical collaterals,^84^ or inputs from other brain areas such as the cortex. Indeed, local inhibitory inputs contributed 7.8% and other extraretinal inputs contributed up to 6.0% of synapses in a dLGN interneuron reconstructed electron-microscopically.^33^ An intriguing question for future research will be whether the visual features of dLGN interneurons are static or are dynamically influenced by extraretinal inputs.

*De novo* computation and inheritance of features from the retina (Figures 3 and 4) in dLGN interneurons parallel the thalamocortical pathway to the primary visual cortex, for which *de novo* computation^85^^,^^86^ and inheritance of features^86^^,^^87^ have also been described. dLGN interneurons are well positioned to contribute to the emergence of novel features or the sharpening and gating of features that are inherited from the retina by the visual cortex.

### A possible role for dLGN interneuron specialization: Feature-selective attention

What function could specialized interneurons fulfill in the dLGN? Extraretinal inputs, such as from the cortex, could modulate—activate or inhibit—individual interneurons encoding specific visual features. Since dLGN interneurons have extensive dendritic arbors in which they display backpropagating action potentials,^47^ changes in interneuron activity are globally distributed via their dendrites across the dLGN. Importantly, the backpropagating spikes evoke widespread GABA release from interneuron dendrites and inhibitory currents in the postsynaptic TCN dendrites.^49^ Together with their feature selectivity, these properties could enable interneurons to perform feature-selective inhibition at the first central stage of vision. Extraretinal inputs activating or inhibiting this feature-selective inhibition could then mediate feature-selective attention, which is thought to gate visual information flow as early as in the dLGN.^88^

The triadic circuit would be beneficial under this hypothesis for the following reasons. Within a triadic motif, a TCN dendrite receives input from the same RGC as the interneuron dendrite, which provides inhibition to the TCN dendrite. Therefore, the triadic arrangement of synapses increases the likelihood that an interneuron inhibits TCNs encoding a similar or the same feature while reducing the probability of inhibiting TCNs encoding distinct features. Such a circuit mechanism ensuring similarity of information content could support the gating of individual visual features by dLGN interneurons, which might be necessary for processes like feature-selective attention.

### The cause and consequence of cell-wide feature selectivity

The finding that the encoded visual features measured within the dendrites and somata of interneurons display high similarity *in vivo* (Figures 6A–6F) is consistent with the existence of backpropagating spikes described *in vitro*.^47^^,^^49^ On the other hand, it would also be consistent with the opposite causality, i.e., the somatic responses resulting from the overall distribution of dendritic inputs. These backwards and forwards causal relationships are not mutually exclusive, especially in a selector model with dominant contribution of few retinal channels with a common visual feature and consequently high pre- and postsynaptic correlation. Together, postsynaptic excitation and backpropagating potentials will determine the local dendritic excitation and response selectivity, which in turn determines the dendritic inhibitory outputs.

*In vitro*, synchronous activation of multiple convergent RGCs is required to evoke active interneuron firing.^49^ In visual stimulation that covers large areas of the retina, different RGCs are activated simultaneously, providing an explanation for the reliable and strong somatic visual responses that we recorded. The heavy attenuation along the interneuron dendrites predicted by computational simulations for subthreshold voltages^35^—and observed *in vitro* using glutamate uncaging^41^^,^^42^—likely underestimated the somatic excitation evoked by multiple convergent inputs.

In addition to dendritic outputs, inhibition could also be distributed via the less-prevalent axonal output synapses of the interneurons, which can be expected to reflect somatic activity and features. Whether these outputs contact dendrites of TCNs with different or similar feature selectivity remains to be shown.

### Ipsilateral versus contralateral responses

Interestingly, binocular dLGN interneurons display an asymmetric preference pattern for retinal input. In both TCNs and interneurons, ipsilateral inputs contain more sustained information. The preference of ipsilateral clusters is consistent with the previous finding that a subset of RGCs (stratifying in the outermost strata) projects ipsilaterally.^89^ The ipsilateral inputs to interneurons, but not TCNs, additionally show an ON preference (Figure S2). While the relative contribution of ipsilateral inputs is small in interneurons (Figure S2) and the functional role of binocular processing in the dLGN is still debated, deserving further experimental study, we argue that the asymmetric arrangement of retinal information in contra- and ipsilateral inputs is not consistent with the hypotheses that ipsilateral inputs to contralateral-input-dominant cells either are developmental remnants that escaped pruning or serve as a silent backup that is unsilenced through activity-dependent mechanisms if the contralateral eye is deprived.^90^

### Superficial versus deep dLGN

Since our electroporations were visually guided, they were biased toward the more superficial/dorsal part of the dLGN, raising the question of how generalizable the results from rabies tracing are across the dLGN. By *in vivo* calcium imaging, on the other hand, we were able to image up to 300 μm from the surface of the dLGN, thus covering large parts of the dLGN (which is ∼500 μm deep). Interestingly, even in the subset of the interneuron population targeted by electroporation, we found a large variety of retinal input specializations, which is consistent with the diversity of features found by *in vivo* imaging. Consistent with the axonal targeting of certain DS RGC types in the superficial/dorsal dLGN,^56^^,^^58^^,^^74^^,^^75^^,^^77^ where tectogeniculate inputs also ramify,^91^^,^^92^^,^^93^ and consistent with superficial DS dLGN neurons showing a preference for motion along the horizontal axis,^79^ both DS interneurons and DS interneurons preferring motion along the horizontal axis were more prevalent in the superficial than in the deep dLGN (Figures 3D and 3E).

Intriguingly, we found a higher percentage of DS inputs to interneurons than to TCNs described previously.^22^ There are two possible explanations for this. First, it is possible that the previous selection of electroporated TCNs in the superficial dLGN (above the ipsilateral projection zone; see Figure S1 of Rompani et al.^22^) was a subset receiving fewer DS inputs than other superficial TCNs, while the interneurons targeted here, in particular due to their large size, are more representative of the entirety of inputs to the superficial part of dLGN. This interpretation would imply that TCNs were sampled from a superficial patch that received fewer DS inputs than the remaining “shell” and indicates that the functional subdivisions of the dLGN go beyond a simple shell-core dichotomy. Second, alternatively or additionally, it is possible that DS retinal inputs could indeed have an overall preference for interneurons over TCNs. In this respect, it should be noted that the previous evidence for dLGN TCNs in the shell being specialized for DS inputs was based on three arguments: first, several DS RGC types preferentially project to the superficial dLGN^56^^,^^58^^,^^74^^,^^75^^,^^77^ (but see also Kay et al.^58^ and Jiang et al.^94^ for DS RGC types that target deeper parts). These studies indicate that the superficial dLGN receives more DS RGC inputs than the deeper dLGN. However, axonal projection patterns do not distinguish postsynaptic cell identity. Second, rabies tracing from upper-layer V1-projecting superficial dLGN neurons labeled almost exclusively DS RGCs.^95^ This rabies tracing utilized G-coated rabies infection in the cortex and relied on G-protein delivery to dLGN neurons via AAV infection, which does not rule out multi-synaptic jumps via interneurons. A third piece of evidence comes from a report that DS neurons in the superficial dLGN prefer horizontal motion.^79^ The percentage of DS neurons in Marshel et al.^79^ was 5% of neurons with DSI > 0.5 in the superficial dLGN, compared to 12.1% of interneurons with DSI > 0.5 (or with *p* < 0.05: 8.9% with DSI > 0.5, 13.5% for DSI > 0.3, 15.6% for DSI > 0.2) among responsive neurons in the upper 100 μm in our study. For comparison, DS RGCs that project to the dLGN make up 17%–35% of all RGCs (DSI criteria 0.2–0.3).^11^^,^^58^^,^^67^^,^^96^^,^^97^ Marshel et al.^79^ used calcium dye for imaging activity, which does not distinguish between TCNs and interneurons. Taken together, both potential causes—a spatial inhomogeneity of DS TCNs and/or DS RGC axons preferentially targeting interneurons—are possible scenarios, and the degree to which they contributed to the surprisingly low number of DS retinal inputs to TCNs compared to interneurons remains to be explored.

## STAR★Methods

### Key resources table

**Table:** 

REAGENT or RESOURCE	SOURCE	IDENTIFIER
**Antibodies**		

Chicken a-RFP	Rockland	Cat# 600-901-379; RRID: AB_10703148
Goat a-ChAT	Millipore	Cat# AB144P; RRID: AB_2079751
Rabbit a-tRFP	Evrogen	Cat# AB233; RRID: AB_2571743
Guineapig a-RFP	Synaptic Systems	Cat# 390 005; RRID: AB_2737051
Rat a-GFP	Nacalai Tesque	Cat# 04404-84; RRID: AB_10013361
Rabbit a-Cart	Phoenix Pharmaceuticals	Cat# H-003-62; RRID: AB_2313614
Rabbit a-Satb1	Abcam	Cat# AB109122; RRID: AB_10862207
Rabbit a-Satb2	Abcam	Cat# AB34735; RRID: AB_2301417
Mouse a-SMI-32	Biolegend	Cat# 801702; RRID: AB_2715852
Rabbit a-GABA	Sigma	Cat# A2052; RRID: AB_477652
Rabbit a-NeuN	Millipore	Cat# ABN78; RRID: AB_10807945
Donkey a-goat Alexa 488	Invitrogen	Cat# A11055; RRID: AB_2534102
Donkey a-goat Alexa 647	Invitrogen	Cat# A21447; RRID: AB_141844
Donkey a-rat Alexa 488	Invitrogen	Cat# A21208; RRID: AB_141709
Donkey a-chicken Cy3	Jackson	Cat# 103-165-155; RRID: AB_2337386
Donkey a-rabbit Alexa 405	Invitrogen	Cat# A48258; RRID: AB_2890547
Donkey a-mouse Alexa 488	Invitrogen	Cat# A21202; RRID: AB_141607
Donkey a-rabbit Alexa 568	Invitrogen	Cat# A10042; RRID: AB_2534017
Donkey a-rabbit Alexa 647	Invitrogen	Cat# A31573; RRID: AB_2536183
Donkey a-guineapig CF568	Biotium	Cat# 20377; RRID: AB_2934264
Hoechst 33342	Thermo Fisher	Cat# H1399; RRID: AB_10626776

**Bacterial and virus strains**		

AAV: AAV-2.1-*syn*-FLEX-splitTVA-EGFP-tTA	Liu et al.^98^	Addgene viral prep # 100798-AAV1; RRID: Addgene_100798
AAV: AAV-2.1-TREtight-mTagBFP2-B19G	Liu et al.^98^	Addgene viral prep # 100799-AAV1; RRID: Addgene_100799
AAV: AAV-2.7m8-CAG-DIO-mWGA-mCherry-WPRE	This manuscript	N/A
Rabies Virus: Envelope-A-coated SADΔG mCherry	Rompani et al.^22^	N/A
Rabies Virus: Envelope-A-coated SADΔG tagRFP	This manuscript	N/A
Rabies Virus: Envelope-A-coated SADΔG Chr2-2A-tdTomato	This manuscript	N/A

**Chemicals, peptides, and recombinant proteins**		

Alexa 594	Thermo Fisher	Cat# A-10438
Fentanyl	Janssen	N/A
Medetomidine	Virbac AG	Cat# QN05CM91
Midazolam	Sintetica	N/A
Buprenorphine, Bupaq P	Richterpharma AG	N/A
Meloxicam, Metacam	Boehringer Ingelheim	N/A
Carprofen, Rimadyl	Zoetis	N/A
Bupivacaine	Sintetica	N/A
Lidocaine HCL	Bichsel AG	N/A
Dexamethasone	Sigma	D2915
Alcaine eye drops	Alcon	N/A
Isoflurane, Attane	Piramal Pharma, Provet AG	Cat# QN01AB06
Coliquifilm	Allergan	Cat# S01XA20
Chlorprothixene	Sigma	Cat# C1671

**Experimental models: Organisms/strains**		

Mouse: GAD67-EGFP	Tamamaki et al.^54^	MGI:3590301
Mouse: GAD2-IRES-Cre	Taniguchi et al.^52^; NIH Neuroscience Blueprint Cre Driver Network et al.	JAX: 010802; RRID: IMSR_JAX:010802
Mouse: vgat-IRES-Cre	Vong et al.^99^	RRID: IMSR_JAX:028862
Mouse: Ai3 (EYFP reporter)	Madisen et al.^53^	JAX: 007903; RRID: IMSR_JAX:007903
Mouse: Ai94(TITL-GCaMP6s)-D	Madisen et al.^100^	RRID: IMSR_JAX:024104
Mouse: CAG-stop-tTA2	Miyamichi et al.^73^	RRID: IMSR_JAX:014092
Mouse: Frmd7tm1a(KOMP)Wtsi	Knouckout Mouse Project (KOMP)	EM: 07372; RRID: IMSR_EM:07372
Mouse: Cart-IRES2-Cre-D	Jackson	JAX:028533; RRID: IMSR_JAX:028533

**Recombinant DNA**		

Plasmid: pAAV-Ef1a-DIO-oG-WPRE	Kim et al.^101^	N/A
Plasmid: pAAV-EF1a-CVS11-GWPRE- hGHpA	Wertz et al.^102^	Addgene plasmid # 67528; RRID: Addgene_67528
Plasmid: pAAV-EF1a-tdTomato- WPRE-hGHpA	Wertz et al.^102^	Addgene plasmid # 67527; RRID: Addgene_67527
Plasmid: pCMMP-TVA800	Wickersham et al.^51^	Addgene plasmid # 15778; RRID: Addgene_15778
Plasmid: pAAV-EF1a-DIO-TVA-WPRE- hGHpA	Rompani et al.	N/A
Plasmid: pAAV-CAG-DIO-mWGA-mCherry-WPRE	Tsai et al.^72^	N/A

**Software and algorithms**		

Custom code (Python) for machine-learning assisted ChAT-band detection	This manuscript	https://github.com/fmi-basel/faim-retina-chatbands; Zenodo: https://doi.org/10.5281/zenodo.11354455
Python 3.6	Python Software Foundation	RRID: SCR_008394; https://www.python.org/downloads/release/python-360/
Keras 2.2.4	François Chollet	https://keras.io/
TensorFlow 1.12.0	Google	RRID: SCR_016345; https://www.tensorflow.org/
Fiji/ImageJ	Fiji/ImageJ	RRID: SCR_002285
MATLAB 2019b/2022b	Mathworks	RRID: SCR_001622; https://www.mathworks.com/products/matlab.html
BioFormats MATLAB toolbox 6.10.0	The Open Microscopy Environment; Linkert et al.^103^	RRID: SCR_000450; https://downloads.openmicroscopy.org/bio-formats/
Probability calculations and Monte-Carlo simulations	Rompani et al.^22^	https://ch.mathworks.com/matlabcentral/fileexchange/61185-presynaptic-mapping
Custom code (MATLAB) to analyze data	This manuscript	https://github.com/fionamuellner/flex-calc-imag; Zenodo: https://doi.org/10.5281/zenodo.11397956

**Other**		

Premium standard wall borosilicate capillary glass pipette	Warner Instruments	Cat# G100-4
Standard Wall Borosilicate Tubing with Filament	Sutter Instruments	Cat# BF100-50-10
Standard Wall Borosilicate Tubing with Filament	Sutter Instruments	Cat# BF100-30-7.5HP
Borosilicate capillary glass pipette	Harvard apparatus	Cat# GC150F-10
Borosilicate glass	Hilgenberg	N/A
3 mm glass coverslip	Warner Instruments	Cat# CS-3R-0
Superglue ultragel	Pattex	Cat# 5843320
Oil injector	Narashige	Cat# IM-9B
Centrifugal filters Ultrafree-MC GV	Millipore	Cat# UFC30GV0S
Dental cement	Paladur	Cat# 128565
Gelfoam gelatin sponges	Pfizer	Cat# 9031508
PMTs	Hamamatsu	Cat# R3896
630nm red LED Array Light Source	Thorlabs	Cat# LIU630A
Olympus IXplore Spin confocal spinning disc microscope system	Olympus	N/A
CSU W1 dual camera T2 spinning disk confocal scanning unit	Yokogawa	N/A
Homogenizer	Visitron	N/A
63x/1.4 Plan Apochromat oil objective	Zeiss	N/A
40x/1.3 Plan Apochromat oil objective	Zeiss	N/A
AxioImager M2 microscope	Zeiss	N/A
AxioObserver	Zeiss	N/A
Edge Cameras	PCA	N/A
Prime 95B cameras	Photometrics	N/A
Laser scanning confocal microscope 720	Zeiss	N/A
Spinning disc confocal microscope	Olympus	N/A
FemtoSMART resonant-galvo scanning microscope	Femtonics	N/A
16× water immersion objective (N16XLWD, 0.8 NA, 3 mm WD)	Nikon	N/A
Hot mirror	Edmund Optics	Cat# 43-453
Eye camera	Imaging Source	Cat# DMK22BUC03
Lens w/Locking Iris & Focus	Imaging Source	Cat# M5018-MP2
Epifluorescence microscope	Olympus	Cat# SZX16
Tunable Ti-Sapphire Laser InSight X3	Spectraphysics	N/A
Axoporator 800A	Molecular Devices	N/A
Manipulator MPC 200	Sutter Instruments	N/A
Objective MPlan N 5×/.1NA	Olympus	N/A
Objective LUMPlanFl 40×/0.8NA	Olympus	N/A
KWIK-CAST	World Precision Instruments	N/A
Stereotactic apparatus	Kopf Instruments	Cat# 1900
P-97 micropipette puller	Sutter Instruments	RRID: SCR_016842
Vibratome VT1000S	Leica	RRID: SCR_016495
ProLong Gold	ThermoFisher	Cat# P36934
Coverslips	Zeiss	Cat# 10474379
Parafilm	VWR	Cat# 52858-000
KLIBA NAFAG irradiated rodent breeding diet	Provimi Kliba AG	Cat# 3302.PM.V20
Ventilated Cages	Tecniplast	Cat# GM 500
Bedding material Lignocel	Rettenmaier & Söhne	Cat# BK8-15
Nesting/Enrichment material	Zoonlab	N/A

### Resource availability

#### Lead contact

Further information and requests for resources and reagents should be directed to and will be fulfilled by the lead contact, Botond Roska (botond.roska@iob.ch).

#### Materials availability

All unique reagents generated in this study are available from the lead contact with a completed Materials Transfer Agreement.

#### Data and code availability

All data reported in this paper will be shared by the lead contact upon request. All original code has been deposited at GitHub and Zenodo and is publicly available as of the date of publication. DOIs are listed in the key resources table. Any additional information required to reanalyze the data reported in this paper is available from the lead contact upon request.

### Experimental model and study participant details

#### Animals

Animals were used in accordance with standard ethical guidelines as stated in the European Communities Guidelines on the Care and Use of Laboratory Animals, 86/609/EEC and FSVO Ordinance on Laboratory Animal Husbandry, the Production of Genetically Modified Animals and Methods of Animal Experimentation (Swiss Animal Experimentation Ordinance) SR 455.163. Experiments were approved by the Veterinary Department of the Canton of Basel-Stadt. Animals for electroporation were 7 females and 6 males, 22 to 60 days old (24–34 days old at rabies injection for the single-cell initiated tracing, Figure S1C), from a GAD67-EGFP line^54^ or a GAD65-IRES-Cre line^52^ crossed to the EYFP-reporter line (Ai3^53^). Animals for imaging were 2–6 months old: 6 males and 6 females from GAD65-IRES-Cre^52^ crossed to Ai94D^53^ and CAG-stop-tTA2,^73^ referred to as wild-type mice; males from this cross were additionally crossed to Frmd7^tm1a(KOMP)Wtsi^ (X-linked mutation)^52^ homozygous females to obtain 5 hemizygous males, referred to as FRMD7™. 2 additional males from vgat-IRES-Cre^99^ crossed with Ai94D and CAG-stop-tTA2 were included as wild-type. 3 heterozygous FRMD7™ females were not included in the wild-type or FRMD7™ mice categories, because they exhibited an intermediate phenotype (Figure S4). Animals for conditional AAV-assisted rabies tracing were 30 male and 13 female, 43 days to 8 months old GAD65-IRES-Cre mice,^52^ of which 23 were from a crossing with GCaMP6s reporter line Ai94D^53^ and CAG-stop-tTA2^73^ and 3 were from a crossing with the EYFP-reporter line (Ai3^53^). 9 control animals negative for Cre were included in this group. Animals for anterograde tracing were 6 male, 3–8 months old Cart-IRES2-Cre-D mice, of which 3 were crossed to the GAD67-EGFP line,^54^ including 1 control animal negative for Cre. Animals were maintained on a 12-h light/dark cycle, fed with irradiated food (KLIBA NAFAG irradiated rodent breeding diet 3302.PM.V20, Provimi Kliba AG) *ad libitum* and autoclaved, chlorinated, and acidified tap water. Mice were kept in individually ventilated cages (GM 500, Tecniplast) with bedding (Lignocel BK8-15, Rettenmaier & Söhne GmbH & Co KG) and nesting/enrichment material (Zoonlab GmbH). Health monitoring was done according to FELASA Guideline 2014.

### Method details

#### Single-cell-initiated rabies tracing

Mice were anesthetized with FMM (fentanyl 0.05 mg/kg, medetomidine 0.5 mg/kg, midazolam 5.0 mg/kg). Dexamethasone (2 mg/kg, Sigma D2915) was injected to prevent an immune reaction. Coliquifilm (S01XA20, Allergan) was applied to the eyes to prevent dehydration. A craniotomy was made on the left hemisphere of the mouse skull. Part of cortex and hippocampus above the left dLGN were aspirated to produce an opening of 3 mm diameter centered on the dLGN. The opening above the dLGN was rinsed with Ringer’s solution (150 mM NaCl, 2.5 mM KCl, 2 mM CaCl_2_, 1 mM MgCl_2_, 10 mM HEPES in ddH_2_O, pH 7.4, 0.2 mm sterile filtered). Gelfoam gelatin sponges (Pfizer 9031508) were used to absorb blood. The animal was placed under a custom-made two-photon microscope equipped with red/green detection channels (Hamamatsu R3896 PMTs) and a red LED light source (630 nm Red LED Array Light Source, LIU630A, Thorlabs). An electroporation solution was made containing 40 μL of intracellular solution (130 mM K-methanesulphonate, 10 mM HEPES, 7 mM KCl, 2 mM Na_2_-ATP, 2 mM Mg-ATP, 0.05 mM EGTA in ddH_2_O, 310 mOsm, pH 7.2), 1.5 μL each of pAAV-EF1a-DIO-TVA-WPRE-hGHpA (in GAD65-IRES-Cre) or pCMMP-TVA800 (in GAD67-EGFP), pAAV-EF1a-CVS11-G-WPRE-hGHpA, and pAAV-EF1a-tdTomato-WPRE-hGHpA (all at a final working concentration of ∼0.1 μg/μL), and 2.5 μL Alexa 594 (1 mM in intracellular solution, A-10438, Thermo Fisher). pCMMP-TVA800 was a gift from Edward Callaway (Addgene plasmid # 15778; http://n2t.net/addgene:15778; RRID:Addgene_15778). The solution was filtered through a 0.2 mm Ultrafree-MC GV Centrifugal Filter (UFC30GV0S, Millipore). In 3/13 animals with the GAD65-IRES-Cre genotype, pAAV-EF1a-CVS11-G-WPRE-hGHpA was replaced by pAAV-Ef1a-DIO-oG-WPRE^101^; however, presynaptic RGC numbers were not increased by oG (70 ± 18 cells with oG versus 105 ± 28 cells with CVS11G; mean ± sem), possibly because transsynaptic labeling was already saturated with CVS11-G. The electroporation solution was loaded into a glass needle (Standard Wall Borosilicate Tubing with Filament, BF100-50-10, Sutter Instruments, resistance 10–30 MΩ) and placed onto the headstage of an electroporation device (Axoporator, 800A, Molecular Devices) mounted on a manipulator (MPC 200, Sutter Instruments). The electrode was placed on the surface of the dLGN using local landmarks (Figure S1A) and a 4× objective (MPlan N 5×/.1NA, Olympus). Green-fluorescent interneurons in the dLGN and the Alexa-filled pipette were visualized by two-photon imaging at 850 nm through a 40× objective (LUMPlanFl 40×/0.8NA Water immersion, Olympus) and electroporated using the settings: voltage = −6-14 V, DC offset = 0, train = 10-1000 ms, frequency = 100 Hz, pulse width 50–500 μs. In 10 animals, only one green-fluorescent neuron was electroporated per animal. Two animals, in which additionally targeted cells were not filled or did not survive electroporation, were also counted as single-cell initiated. In one animal, 4 GFP-positive cells were electroporated; this animal was included only when estimating the overall distribution of presynaptic RGC types (Figure 2H). After retraction of the pipette, the surgical window was filled with KWIK-CAST (World Precision Instruments) or a 3-mm glass cylinder sealed with a 3-mm coverslip (see “calcium imaging”), was implanted. The mouse received buprenorphine (0.1 mg/kg) for postoperative analgesia and was placed in a heated cage to recover. Up to 7 days after the electroporation, the window/plug was removed (pharmacological treatment as above), a pulled glass needle (Premium Standard Wall Borosilicate, Model G100-4, Warner Instruments) was cut at the tip (1–3 MΩ) and loaded with EnvA-coated SADΔG-rabies. The EnvA-coated SADΔG-rabies virus was produced as described previously^22^^,^^102^ and expressed either mCherry^22^ or tagRFP (generously provided by Karl-Klaus Conzelmann). The viral titer ranged from 10^8^ to 10^12^ plaque-forming units/ml. In order to minimize dilution of the rabies virus, Ringer’s media from the dLGN surface was removed. Rabies virus (250 nL–1.2 μL) was injected within 200 μm of the electroporated cell. To allow virus diffusion within the tissue, the pipette was left in place for >10 min before retraction. Mice were sacrificed 10–11 days after rabies injection.

#### Conditional AAV-initiated rabies tracing

Mice were anesthetized with FMM (fentanyl 0.05 mg/kg, medetomidine 0.5 mg/kg, midazolam 5.0 mg/kg). Dexamethasone 2 mg/kg (Sigma D2915) was injected to prevent an immune reaction. Coliquifilm (S01XA20, Allergan) was applied to the eyes to prevent dehydration. The animal was placed in a stereotactic frame (#1900, Kopf Instruments). A 1:1 mix of bupivacaine 5 mg/mL and lidocaine 2 mg/mL was injected subcutaneously at the site of skin incision. A small craniotomy was made on the left hemisphere of the mouse skull. 30–100 nL of AAV-2.1-*syn*-FLEX-splitTVA-EGFP-tTA (final dilution 1:160 in PBS) and AAV-2.1-TREtight-mTagBFP2-B19G (final dilution 1:15)^66^ were slowly injected at 2.0–2.25 posterior of bregma, 1.84–2.15 lateral from midline and 2.25–2.45 depth from dura with a pulled glass needle (Sutter instruments, B100-30-7.5HP, ID 0.30 mm). The mouse received buprenorphine 0.05 mg/kg and meloxicam 5 mg/kg for postoperative analgesia. Anesthesia was antagonized by revertor 2.5 mg/kg and flumazenil 0.5 mg/kg. 7–8 days after the AAV injection, the surgery was repeated (pharmacological treatment as above) and 70–300 nL of EnvA-coated SADΔG-Chr2-2A-tdTomato-rabies were injected at the previously injected coordinates. AAV-2.1-*syn*-FLEX-splitTVA-EGFP-tTA and AAV-2.1-TREtight-mTagBFP2-B19G were a gift from Ian Wickersham (Addgene viral preps # 100798-AAV1 and 100799-AAV1; RRID:Addgene_100798 and RRID:Addgene_100799; http://n2t.net/addgene:100798 and http://n2t.net/addgene:100799). The viral titer was 10^9^ plaque-forming units/ml for the rabies and 10^13^ plaque-forming units/ml for the AAVs before dilution. Mice were sacrificed 7–10 days after rabies injection. Center and size of infection were examined in coronal sections (see Immunohistochemistry below). From 32 double-injected, Cre-positive samples, *n* = 13 samples with infection center localized within the dLGN and labeled cells in the retina were included and the corresponding retinas evaluated. The negative controls (*n* = 9 Cre-negative double-injected, *n* = 2 Cre positive with no AAV, only rabies injected) did not contain labeled cells.

#### Anterograde tracing

Mice were anesthetized with 1.5–3% isoflurane (v/v in 95% O_2_). Alcaine 0.5% eyedrops were applied before scleral puncture. 1–2 μL of AAV-2.7m8-CAG-DIO-mWGA-mCherry-WPRE^72^ were injected intravitreally to the right eye with a pulled glass needle (Harvard Apparatus, GC150F-10, ID 0.86 mm). The mouse received Carprofen 5 mg/kg for postoperative analgesia. The viral titer was 10^11^ plaque-forming units/ml. Mice were sacrificed 30 days after injection. The negative control (*n* = 1 of 6) did not contain labeled cells.

#### Immunohistochemistry

After euthanasia, eyes and brains were harvested and fixed in 4% PFA overnight at room temperature (RT) or 2 days at 4°C. Retinas and brains were washed 3x in PBS, transferred to 30% sucrose in PBS (w/v), allowed to sink and then subjected to 3 freeze-thaw cycles. Retinas were dissected and brains cut into 200 μm coronal sections with a vibratome (Leica VT1000S). They were again washed 3x in PBS and incubated in heavy blocking solution (10% NDS, 1% BSA, 0.5% Triton X-100, 0.01% sodium azide in PBS) for 1 h (retina) or 3h (brain) at RT. Primary antibodies (goat a-ChAT Millipore AB144P 1:200, chicken a-RFP Rockland 600-901-379 1:1000 (for mCherry-rabies) or rabbit a-tRFP Evrogen AB233 1:1000 (for tagRFP-rabies) or guineapig a-RFP Synaptic Systems 390 005 1:2000 (for tdTomato-rabies or mWGA-mCherry-AAV), rat a-GFP Nacalai Tesque 04404-84 1:1000 (retina) or 1:2000 (brain), rabbit a-Cart Phoenix Pharmaceuticals H-003-62 1:2000, rabbit a-Satb1 Abcam AB109122 1:1000, rabbit a-Satb2 Abcam AB34735 1:1000, mouse a-SMI-32 Biolegend 801702 1:2000, rabbit a-GABA Sigma A2052 1:2000, rabbit a-NeuN Merck ABN78 1:1000) were prepared in light blocking solution (3% NDS, 1% BSA, 0.5% Triton X-100, 0.01% sodium azide in PBS) and retinas/brain slices treated for 3–14 days at RT. Retinas/brain slices were then washed 3x with PBS. Secondary antibodies (1:200 for retinas, 1:500-1000 for brain slices, donkey a-goat Alexa 488 A11055 or donkey a-goat Alexa 647 A21447 Invitrogen, donkey a-chicken Cy3 Jackson F03-165-155, donkey a-rabbit Alexa 405 A48258 or a-rabbit Alexa 568 A10042 or a-rabbit Alexa 647 A31573 Invitrogen, donkey a-rat Alexa 488 A21208 Invitrogen, donkey a-mouse Alexa 488 A21202 Invitrogen, donkey a-guineapig CF568 Biotium 20377, 1:2000 Hoechst 33342) were prepared in light blocking solution and retinas were treated for 1–2 h at RT, brains for 2-12 h at RT. After 3x washing in PBS, the retinas and brains were mounted with ProLong Gold (P36934 ThermoFisher). For retinas, strips of Parafilm (52858-000 VWR) were placed at the coverslip (D = 0.17 mm, Zeiss 10474379) borders to prevent tissue compression.

#### Confocal microscopy

Confocal image stacks for single-cell-initiated rabies tracing data were acquired using spinning disc microscopes with a CSU W1 dual camera T2 spinning disk confocal scanning unit (Yokogawa), a homogenizer (Visitron), 63x/1.4 or 40x/1.3 Plan-Apochromat oil objectives (Zeiss), and an MS2000X,Y stage with a Z-Piezo drive (ASI). The upright system was built on an AxioImager M2 microscope (Zeiss) and equipped with two Edge cameras (PCO). The inverted system was built on an AxioObserver (Zeiss) and equipped with two Prime 95B cameras (Photometrics). Images had a pixel size of ∼0.2 μm in xy, 0.25 μm in z. Appropriate illumination and filters were used for Alexa 488 (ChAT) and Cy3/Alexa 568 (RGCs) and a subset of images acquired on a Zeiss LSM 720. Sub-micrometer z-offsets between the red and green channels were detected and compensated post hoc. All other confocal image stacks were acquired with an Olympus Ixplore Spin confocal spinning disc microscope system. We manually annotated the center of the presynaptic RGC clusters and quantified their eccentricity (between 0° at the optic nerve and 115° at the peripheral border^104^).

#### ChAT-band detection

To generate training data, ChAT bands were manually annotated as two lines in the yz-projections of a subset of confocal stacks. These data were used to train a U-Net,^55^ implemented in Python using Keras/TensorFlow, in order to detect ChAT bands in 3D stacks. The output was provided as a 3D probability stack. Subsequent analysis was performed in MATLAB (R2019b/R2022b, Mathworks). From the 3D probability stack, the two ChAT bands were automatically detected in yz-projections as lines of local maxima, resulting in a 2D (xy) matrix of z-coordinates for each band. Correct assignment of the ChAT bands was manually verified and curated if necessary.

#### Classification of RGCs

The z-coordinates of the ChAT bands were used to artificially flatten the image stacks by assigning each pixel its relative position to the ChAT coordinates in z, set to relative position 0 and 1, and interpolating the stack at z-increments of 0.025. 10 strata were defined at regular intervals of 0.25, with strata 3 and 7 centered around the OFF- respectively ON-ChAT coordinates. For the red channel (of labeled RGCs), a maximum z-projection was pseudo-colored with respect to the stratum in which the pixel with maximum brightness was located (Figures 2C, 2F, 2G, S1F, and S2A). Additional maximum projections per stratum of both channels aided the classification (for example to evaluate co-fasciculation of dendrites with the ChAT processes). Based on these visualizations, individual RGCs were manually classified by the stratification of their terminal dendrites. Type 12 included cells with stratification in strata 1 and/or 2. If the dendrites also displayed asymmetry characteristic of JAM-B, the cells were classified as 12_asym. Type 89 included the previous types 89_big and 89_PV1,^22^ as well as other cells stratifying in strata 8 and/or 9 and/or 10. Type 189 included the previous types 189_big and 189_small,^22^ as well as other cells stratifying in 1, 2 and 8 and/or 9 and/or 10. Type 4 included the previous types 4_giPV5, 4_PV5, 4_PVX.^22^ Type 37 cells co-fasciculated with the ChAT processes in 3 and 7. Type 7 co-fasciculated with the ON-ChAT stratum 7, with only minor branches to stratum 3 (see examples in Figure 2G). Cells stratifying in 689 were not found amongst the cells presynaptic to dLGN interneurons. DS ratios (Figure 4A) were measured as the ratio of presynaptic DS RGCs (defined as co-fasciculating with the ChAT bands, including or excluding RGCs stratifying in strata 1 and 2 and displaying asymmetric dendrites characteristic of JAM-B cells) relative to all classified RGCs presynaptic to a single dLGN interneuron. Of *n* = 1253 RGCs presynaptic to dLGN interneurons, *n* = 688 could be classified. This included the *n* = 638 classified out of *n* = 1153 RGCs labeled by *n* = 12 single-cell-initiated rabies tracing, plus one experiment with 4 electroporated interneurons. Data for TCNs refer to the *n* = 245 classified out of *n* = 507 total RGCs previously reported.^22^

For AAV-initiated rabies tracings, we morphologically classified 25 Cart-positive RGCs presynaptic to dLGN interneurons (*n* = 3 animals) as well as an unbiased sample (agnostic of Cart-label) of 104 RGCs presynaptic to dLGN interneurons (*n* = 6 animals), out of which 20 were Cart positive. RGCs were classified as bistratified if they stratified in at least one ON and one OFF stratum.

#### Probabilistic modeling

Monte Carlo simulations were performed in MATLAB (R2019b/R2022b, Mathworks) to simulate the null hypothesis that presynaptic RGCs were randomly drawn from a given distribution of cell types. To obtain conservative estimates of specialization, we assumed that the distribution of cell types projecting to the dLGN is not uniformly random, but reflected in the overall distribution of cell types projecting to TCNs (which itself is specialized^22^) and the overall distribution of cell types presynaptic to dLGN interneurons (Figure 2H). To compare specialization between dLGN interneurons and TCNs, we estimated the overall distribution of cell types by the empirical distributions p_TCN_ and p_IN_ found for TCNs, respectively interneurons, and calculated a weighted average (p_TCN_ ⋅ W_TCN_ ⋅ N_TCN_ + p_IN_ ⋅ W_IN_ ⋅ N_IN_), with N_TCN_ = 20, N_IN_ = 96 being the average empirical numbers of presynaptic RGCs per TCN/interneuron and W_TCN_ = 0.8, W_IN_ = 0.2 to account for the higher prevalence of TCNs. From this distribution, the expected number of cell types present in any given number of presynaptic cells and its standard deviation were simulated. Specialization *Z* scores were calculated as the difference between empirically found and expected number of cell types divided by the simulated standard deviation; the number of classified RGCs was taken as the total number of cells for this calculation. Our method quantifies which dLGN cells are significantly more specialized than what would be expected based on overall cell-type specialization within the targeted region of the dLGN, which is superficial and anteromedial above the ipsilateral projection zone (Figure S1A). The input specialization might nevertheless reflect axonal selectivity on a smaller spatial scale.

#### Simulations how the number of cell types influences the specialization *Z* scores

Increasing the number of cell types in the classification increases heterogeneity of both the sample and the reference population, and therefore it does not necessarily decrease the specialization *Z* score. We performed simulations, how the number of cell types influences the specialization *Z* scores (Figure S6D–S6E). In summary, two effects prevail when increasing the number of classified cell types:

(1) A specialization toward a subset of RGCs can become insignificant, because the statistical power decreases with number of classified cell types.
(2) Additional specializations to subsets of RGC types can appear, which were previously hidden.

To illustrate these effects in numbers: Let us assume 40 equally distributed cell types and simulate specialization for 50 presynaptic RGCs. Our Monte Carlo simulation then indicates that up to 25 cell types would be expected by chance, less than 25 cell types would indicate specialization.

If an interneuron would now be specialized toward the 26 OFF and ON-OFF types amongst the 40 types, completely lacking all ON types, this specialization would not be detected (*Z* score = −1.4). If cell types are meaningfully pooled with a factor of 2, not mixing ON-OFF categories, and we repeat the simulation, the 13 pooled cell types are now detected as significant specialization (*Z* score = −5.0).

If an interneuron would be specialized toward type #1-#20 of 40 types, this specialization is detected as highly significant without pooling (*Z* score = −4.5). If the first 20 cell types are pooled with a factor of 2, pooling each type with another type from #21-#40 (#1 and #21, #2 and #22, and so forth), the specialization would no longer be significant (*Z* score = 1.4) and therefore hidden.

Therefore, we pooled cell types with similar stratification patterns, without pooling across the functional categories we aimed to interpret (sustained/transient, ON/OFF, DS/non-DS).

#### *In vivo* two-photon calcium imaging

Surgery was performed (see “Conditional AAV-initiated rabies tracing”, same pharmacological treatment) to obtain a 3-mm cranial window over the left dLGN, with overlying parts of cortex and hippocampus removed. A custom-made cylinder of borosilicate glass (OD 3 mm, ID 2.55 mm, length 1.6 mm), sealed with a 3-mm glass coverslip (CS-3R-0, Warner Instruments), was implanted and fixed to the skull with superglue (Pattex Ultragel). A custom-made aluminum headplate was attached to the skull with superglue and dental cement (Paladur). Two-photon calcium imaging was performed after recovery from the surgery, between d1 and d41 after window implantation (mean ± SD: 17 ± 15 days across all data). 8.08% of all data were acquired on d1, half of them anesthetized (4.04%) and half of them awake (4.04%). We performed calcium imaging in 22 mice (5 FRMD7™ hemizygous, 3 heterozygous, and 14 wild-type). All mice were recorded under anesthesia, except 1 wild-type animal which was only recorded awake. Additional awake recordings were performed in 9 of the other 13 wild-type mice. Mice were head-fixed via the headplate. For anesthetized recordings, mice were injected with 1.25 mg/kg chlorprothixene prior to recording, lightly anesthetized with ∼0.5% isoflurane during recording and kept on a heating pad. For awake recordings, mice were accustomed to and later imaged while being head-fixed and freely running on a wheel. An LED screen (52.5 cm wide, 29.5 cm high, 15 cm distance) was placed in front of the right eye. The glass cylinder was filled with ddH_2_O prewarmed to 38°C. Two-photon imaging was performed using a FemtoSMART resonant-galvo scanning microscope equipped with a 16× Nikon water immersion objective (N16XLWD, 0.8 NA, 3 mm WD), which was warmed and light-shielded. Data were acquired at 920-nm illumination with up to 60 Hz, 0.4–1.8 μm per pixel, up to 475 × 475 μm field of view. For visual stimulation except receptive field mapping, white-black gratings were shown that moved in 8 different directions with 3 different velocities (400/1200/2400 μm/s on the retina, white bar width 10°, 40° per cycle, presented for 4 cycles: 12 s for 400 μm/s, 4 s for 1200 μm/s, 2 s for 2400 μm/s velocity, corresponding to 0.33/1/2 Hz). A gray screen with the same average (25%) luminance was shown between moving gratings. For receptive field mapping, the screen covering 116° in width and 86° in height of the visual field was divided in equal squares of on average 8° width. White squares were flashed for 0.6 s on a black background at pseudo-randomized positions with 0.6 s in between subsequent flashes. Receptive fields were recorded during anesthesia. A hot mirror (Edmund Optics, #43–453) was placed in between the mouse, pupil position was tracked with a video camera (Imaging Source, DMK 22BUC03 with M5018-MP2 focus) and confirmed to be stable.

To obtain anatomical xyz coordinates of individual dLGN interneurons, we aligned the maximum projection across time of each dataset to z-stacks across depth, which we in turn aligned to relative anatomical dLGN coordinates by setting the angle of the axon tract above the dLGN to 0° (mediolateral axis) and defining an origin at the anteromedial tip of the dLGN. The xy coordinates of *n* = 1917 of 2316 interneurons, all recorded in the same region (Figure S1I) were mapped. The z-depth refers to depth below the optic tract (cells start at 0 μm), which is ∼50 μm below the dLGN surface.

#### Data analysis

Data were processed by custom-written software in MATLAB (R2019b/R2022b, Mathworks). Bidirectional scanning artifacts were corrected and rigid motion-correction was performed. Data were filtered by a moving average filter of 500 ms length. Regions of interest (ROIs) were automatically detected based on local correlations after filtering, briefly: A matrix of local correlations was calculated. ROIs were initiated at local maxima of the correlation matrix and extended to neighboring pixels if a correlation threshold was exceeded, which depended both on local correlations of the seeding pixel and the local background correlations. Local background was defined as the filtered average signal of the 5 darkest pixels within 15 μm distance. Local background was subtracted for all ROIs (even though it contained visible signals only in a few very densely labeled regions). For each recording (3–15, on average 4.4 repetitions of moving gratings in 8 different directions at 3 different velocities), ROIs corresponding to putative somata were manually selected from an overlay of the 95^th^ percentile projection and the local correlation matrix. For all putative somata, local correlation matrices and maximum projections over the frames with highest activity at this ROI were inspected and discarded if they were not indicative of a soma (Figure S6A). To avoid duplication of cells, corresponding imaging regions recorded consecutively or on different days were aligned, matching ROIs were determined (Figure S6B) and the data for each ROI was concatenated, respectively the dataset with the highest SNR was selected. SNR was defined as maximum absolute response amplitude divided by the standard deviation of the baseline. ROIs were excluded if the SNR did not exceed 2.5, which excluded 9.8, 7.8, 11.75, and 6.9% of ROIs in wild-type anesthetized, wild-type awake, FRMD7tm heterozygous, and FRMD7tm hemizygous mice. Baseline was defined as median signal within 1.75–0.25 s before stimulus onset. The median response of raw signals for each stimulus was determined after baseline subtraction. The median response was filtered, its positive response P_i_ (maximum), negative response N_i_ (-minimum), and average across the stimulation period were calculated. A response was counted as suppressed if the average was below baseline. If >50% of the 24 average responses were negative, the cell was classified as SbC. For each velocity, the direction- and orientation-selectivity indices were calculated from the 8-dimensional tuning vectors P_1-8_ and N_1-8_ (after setting negative values to zero) to directions ϑ_1-8_ based on circular variance:

$$\text{DSI}=\frac{\sqrt{{\left(\sum P_{i\cdot }\;\sin\;\vartheta_{i}\right)}^{2}+{\left(\sum P_{i\cdot }\;\cos\;\vartheta_{i}\right)}^{2}}}{\sum P_{i\;}}{,\text{DSI}}_{\text{neg}}=\frac{\sqrt{{\left(\sum N_{i\cdot }\;\sin\;\vartheta_{i}\right)}^{2}+{\left(\sum N_{i\cdot }\;\cos\;\vartheta_{i}\right)}^{2}}}{\sum N_{i\;}}$$

$$\text{OSI}=\frac{\sqrt{{\left(\sum P_{i\cdot }\;\sin\;{2\vartheta }_{i}\right)}^{2}+{\left(\sum P_{i\cdot }\;\cos\;{2\vartheta }_{i}\right)}^{2}}}{\sum P_{i\;}}{,\text{OSI}}_{\text{neg}}=\frac{\sqrt{{\left(\sum N_{i\cdot }\;\sin\;{2\vartheta }_{i}\right)}^{2}+{\left(\sum N_{i\cdot }\;\cos\;{2\vartheta }_{i}\right)}^{2}}}{\sum N_{i\;}}$$

If the SNR of the positive response was ≤2.5, DSI was set to zero. If the SNR of the negative response was ≤2.5, DSI_neg_ was set to zero. Preferred directions/orientations were defined as the angle of the population vector (complex phase for direction, half the complex phase for orientation) and were corrected for the 20° angle between the mouse eye main axis and screen horizontal axis (Figure S6C). All polar plots shown display responses as ΔF/F_0_, with F_0_ defined as smallest baseline of the 24 average responses. For speed-specific plots, SNR and percentage suppressed by contrast were calculated per velocity, and only data with SNR > 2.5 were included. For calculating speed-independent DSI, respectively OSI per interneuron, the speed which evoked the maximum response was selected (which could be a different speed for DSI_pos_ and DSI_neg_). For calculating speed-independent correlations of dendritic with somatic responses, the speed which evoked the maximum response (positive or negative) was selected. We additionally calculated a significance metric (*p* value) for all four selectivity indices (denoted pDS, pOS, pDS_neg_, pOS_neg_) by randomly permuting individual trials across stimulus orientations, and we considered as selective only interneurons for which the index was larger than 95% of the shuffled control data (*p* < 0.05). Since there was considerable overlap between features (Figure S3E) and individual interneurons could be significantly selective for feature combinations, we defined a hierarchy (DS, OS, DS_neg_, OS_neg_, SbC, broad from high to low), in order to assign each interneuron to a unique category (Figures 3C–3E, Figure S3E,G). This way interneurons with DSI > 0.3, pDS < 0.05 are categorized as DS, even if they additionally display a significant OS, DS_neg_, OS_neg_, or SbC feature. Broadly selective interneurons (broad) were defined as having DSI < 0.15 and OSI < 0.15 and not falling into any other category. The percentages of the individual categories, permitting overlap between them, were higher than indicated in Figure 3E: 10.1% OS, 6.3% DS_neg_ and 5.5% OS_neg_ in the upper 100 μm of the dLGN.

For receptive field mapping, the raw data of 4–7 (mean 6) trials was temporally aligned for each stimulus position at stimulus onset, interpolated with 33 Hz, averaged, and filtered with a running average over 150 ms. The time intervals of 200-75 ms before stimulus onset respectively offset were defined as baseline for ON respectively OFF responses. To account for biphasic responses, the maximum or minimum amplitude after baseline subtraction (whichever had larger absolute value) during the flash (ON response) respectively between subsequent flashes (OFF response) was defined as response amplitude, excluding ±75 ms around stimulus on- and offset to avoid filtering artifacts. The single trial data was randomly permuted 10^4^ times and corresponding ON and OFF response maps were calculated as shuffling control. For each 2D map, the local correlation was defined as the maximum correlation of the map with itself, shifted by one pixel in horizontal, vertical, or oblique directions. Receptive fields were considered significant if the local correlation of the map was above 95% of the local correlations of the shuffling controls (*p* < 0.05). The 2D maps of ON and OFF responses with significant receptive fields were filtered by a 3 × 3 running average. For each 2D map, the receptive field was defined as the connected region around the maximum pixel with pixel values above half maximum. Receptive field size was calculated as the area within this region. Receptive field position was calculated as the center of mass of the filtered 2D map within this region. For cells with significant ON and OFF receptive fields (ON-OFF), the area and center of mass of the sum of both receptive fields was calculated. For 53.6% (120/224) interneurons, significant receptive fields were detected (12.5% ON-OFF, 21.9% ON, 19.2% OFF). Similar results were obtained for averages instead of maximum/minimum amplitudes, both with local correlation *p* values and *p* values obtained by a one-way ANOVA applied to single trial average responses, but the percentage of significant receptive fields was lower in both cases (51.8% with 1366 degrees^2^ mean area, respectively 47.8% with 1373 degrees^2^ mean area). To obtain interpolated retinotopic maps (Figure 6C, right panels), a 300 × 300 μm mask with a central 2D Gaussian with σ = 75 μm was added for each interneuron at its position in the map as weight, and a weighted average of azimuth, respectively elevation, of all interneurons with significant receptive field (*p* < 0.05) was calculated at each map coordinate.

#### Dendritic analysis

For all recordings (including the awake condition and recordings in FRMD7™ animals), the overlay of the 95^th^ percentile projection and the local correlation matrix were inspected for cells with dendrites visible in the same imaging plane. From the automatically detected ROIs, dendrites belonging to a soma were manually annotated. In ambiguous cases, for example to distinguish smaller bifurcations from other crossing dendrites, the overall signals (Figure 6A, middle panel) were inspected for similarity. Only compartments with SNR > 2.5 were included in further analysis. The morphology of SbC interneurons, presumably due to their higher baseline activity, could be reconstructed over the longest distances (up to 300 μm). Distances were measured as Euclidean distance between ROI centers, providing a lower estimate for the path length.

### Quantification and statistical analysis

#### Statistics

Non-parametric tests (Mann-Whitney U test, Wilcoxon signed-rank test) were applied for all comparisons of two groups. To compare more groups, we applied the non-parametric Kruskal-Wallis test, followed by post hoc sign-tests for all groups with Bonferroni-Holm correction. Fisher’s exact test was applied for 2x2 contingency tables. Chi-squared test was applied for comparing larger contingency tables, followed by post hoc Fisher’s exact tests for each category against all others with Bonferroni-Holm correction. For the comparison of continuous distributions, Kolmogorov-Smirnov test was applied. To test whether the distribution of presynaptic cell numbers across the two eyes deviated from a random binomial distribution, the *p* value was derived from the symmetric confidence intervals of the Monte-Carlo simulated distributions of the given test-statistics (absolute difference between ipsi- and contralateral cell counts, Figure S1E). The binomial simulation was performed for *p* = 0.87, the average fraction of contralateral cells. For multiple comparisons (Figures 2D, S2J, and S4), the *p* values were Bonferroni-Holm corrected. Pearson correlation coefficients are provided to quantify correlations between response vectors (Figures 6B and 6C). Spearman rank correlation was used to quantify the non-linear distance-dependence of correlation coefficients (Figure 6C).

## Supplemental Information

Document S1. Figures S1–S6, related to Figures 1, 2, 3, 4, and 6 and STAR Methods

Document S2. Article plus supplemental information
