# Supplementary material for: Individual thalamic inhibitory interneurons are functionally specialized toward distinct visual features
Source: Neuron. 2024 Aug 21;112(16):2765–2782.e9. doi: 10.1016/j.neuron.2024.06.001 (PMC11348917; doi:10.1016/j.neuron.2024.06.001)
Supplement: Document S1. Figures S1–S6, related to Figures 1, 2, 3, 4, and 6 and STAR Methods [file mmc1.pdf]

Neuron, Volume *112*

Supplemental information

Individual thalamic inhibitory interneurons are functionally specialized toward distinct visual features

Fiona E. Müllner and Botond Roska

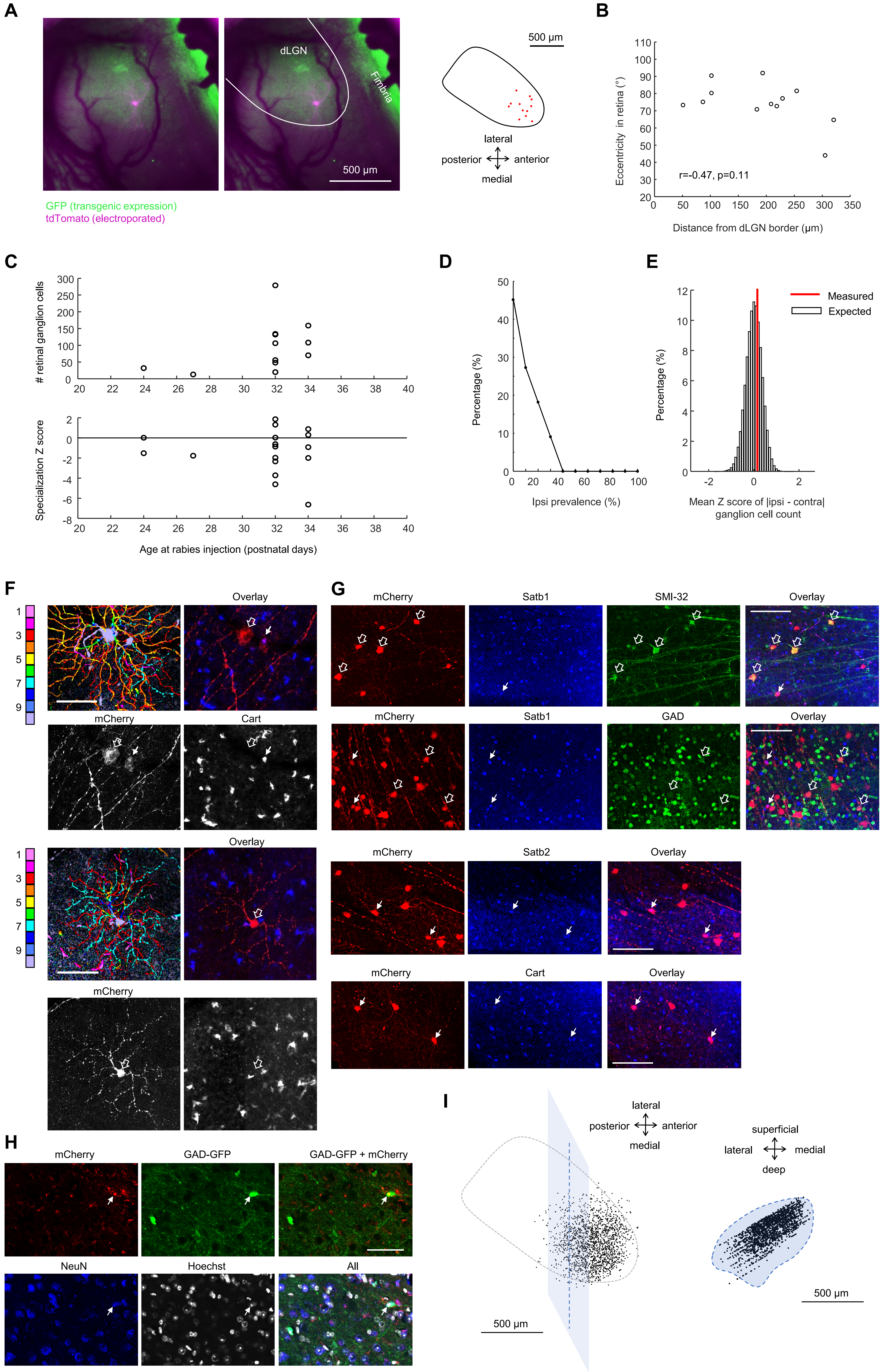
A
dLGN
Fimbria
500 µm
GFP (transgenic expression)
tdTomato (electroporated)
lateral
posterior
anterior
medial
B
Eccentricity in retina (°)
r=-0.47, p=0.11
Distance from dLGN border (µm)
C
retinal ganglion cells
Specialization Z score
Age at rabies injection (postnatal days)
D
Percentage (%)
Ipsi prevalence (%)
E
Measured
Expected
Percentage (%)
Mean Z score of |ipsi - contra| ganglion cell count
F
Overlay
mCherry
Cart
G
mCherry
Satb1
SMI-32
Overlay
GAD
Satb2
Cart
H
mCherry
GAD-GFP
GAD-GFP + mCherry
NeuN
Hoechst
All
I
lateral
posterior
anterior
medial
superficial
lateral
medial
deep
500 µm

Supplemental Figure 1: Method details of single-cell-initiated rabies tracing

Related to Figure 1

A: To target interneurons in the dLGN by single-cell electroporation, the dLGN was visually identified by the following anatomical landmarks established by intravitreal CTB-injections: (1) 3D-curvature of the thalamic surface and (2) blood vessel pattern provide a putative location, which is confirmed by (3) GFP-expression of genetically targeted GABAergic interneurons which have a higher density in the dLGN compared to surrounding structures. Green: native fluorescence of the GABAergic interneurons. Magenta: tdTomato expression of the electroporated interneurons at day 3. Left panel shows an epifluorescence image though the implanted window. Middle panel indicates the scaled outlines of the dLGN in white along the border of green expression and the fimbria. Right panel shows the positions of all electroporated interneurons (red) with respect to the dLGN outlines (black).

B: Eccentricity of the presynaptic RGC cluster center in degree, in relation to the distance of the postsynaptic interneurons from the dLGN borders.

C: Measured number of presynaptic RGCs (upper panel) and specialization Z scores (lower panel) plotted against the age of mice at rabies injection for all single-cell-initiated rabies tracings.

D: Histogram of ipsilateral prevalence, defined as the percentage of presynaptic RGCs found in the ipsilateral retina.

E: The simulated distribution of Z scores for the absolute difference in RGC count between ipsilateral and contralateral is shown in black. The Z score for the measured value |ipsi-contra| is indicated by a red line.

F: Example immunohistochemistry images with Cart antibody for morphologically classified RGCs labeled by single-cell-initiated rabies tracing from dLGN interneurons. Upper four panels: one Cart-negative type 4 and one Cart-positive type 37 RGC with intermingled dendrites. Lower four panels: one Cart-negative type 37 RGC. On each four panels group: Upper left panel: top-down maximum projection with pseudo-colored dendrites according to their stratification (see Figure 2). Upper right panel: Overlay of mCherry (from rabies, red) and Cart (blue) signals. Lower left panel: mCherry. Lower right panel: Cart. Full arrows: Cart-positive, open arrows: Cart-negative. Scalebars: 50 µm.

G: Example immunohistochemistry images with antibodies against mCherry, Satb1, Satb2, Cart, SMI-32 and GFP for GAD-GCaMP6s. Each row shows the zoom-in to one retina, in which RGCs presynaptic to dLGN interneurons are labeled by AAV-initiated rabies tracing. Full arrows: Satb1/Satb2/Cart-positive, open arrows: SMI-32/GAD-positive. Scalebars: 100 µm.

H: Example immunohistochemistry images of dLGN with antibodies against mCherry, NeuN and GFP for GAD-GFP. Direction-selective RGCs in Cart-IRES2-cre animals were infected with an AAV expressing mWGA-mCherry, which anterogradely labels a subset of dLGN neurons, including interneurons. Full arrow: dLGN interneuron double-positive for mCherry and GAD-GFP. Scalebars: 50 µm.

I: Anatomical position of dLGN interneurons from which calcium imaging was performed (Figure 3-6). Left panel: positions in the horizontal plane in top-down projection. Blue line and trapezoid indicate a coronal section at -2 mm from bregma, outlined on the right. Right panel: positions in a coronal projection, rotated to match the angle of the implanted window. Horizontal and coronal outlines were estimated from Paxinos atlas[S1]. xyz positions were mapped for n=1917 of 2316 interneurons recorded in 14 wild-type, 5 hemizygous $FRMD7^{tm}$ und 3 heterozygous $FRMD7^{tm}$ mice; all 2316 were recorded in the region indicated.

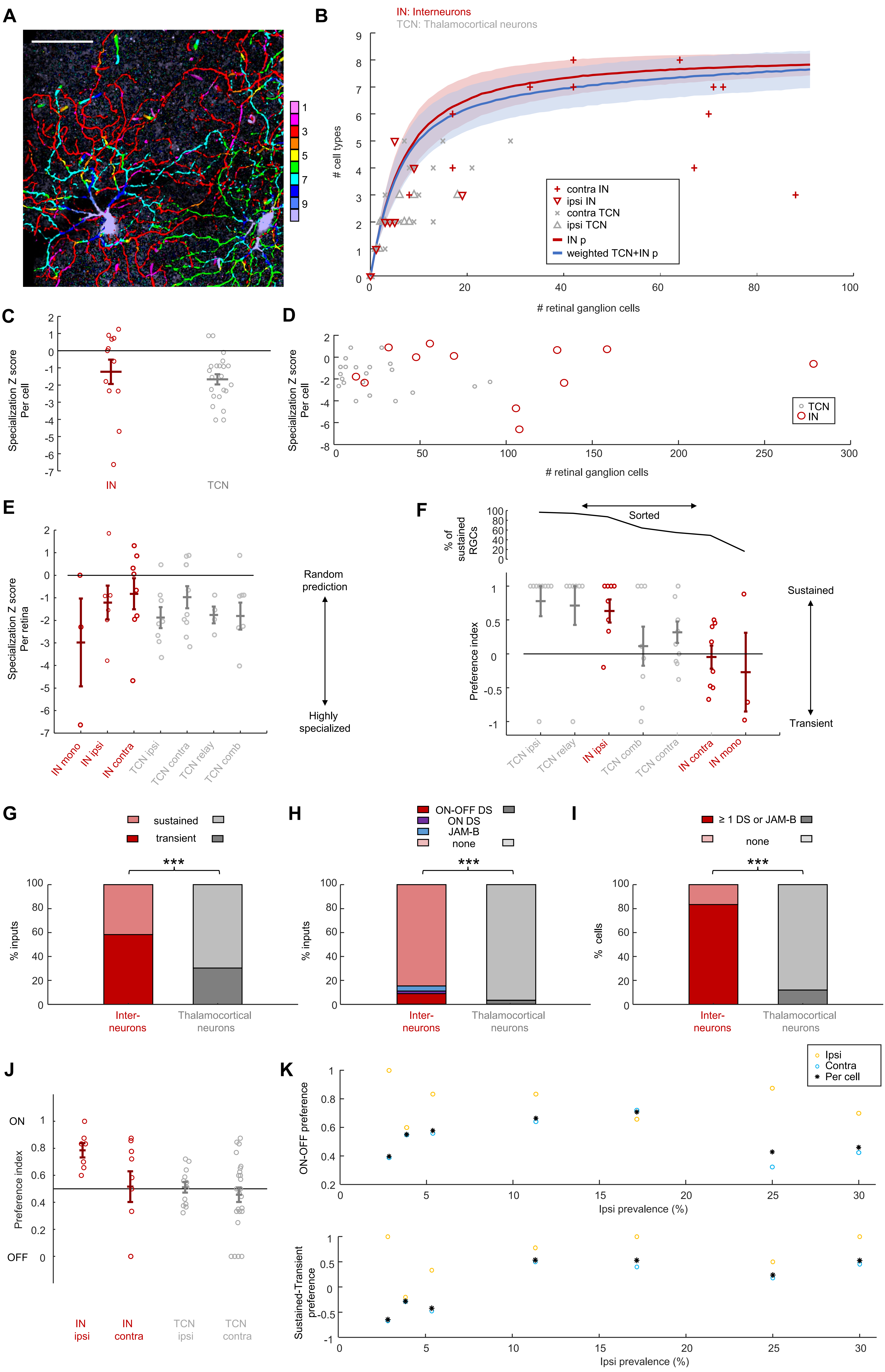
A
1
3
5
7
9
B
IN: Interneurons
TCN: Thalamocortical neurons
cell types
retinal ganglion cells
contra IN
ipsi IN
contra TCN
ipsi TCN
IN p
weighted TCN+IN p
C
Specialization Z score
Per cell
IN
TCN
D
Specialization Z score
Per cell
retinal ganglion cells
TCN
IN
E
Specialization Z score
Per retina
Random prediction
Highly specialized
IN mono
IN ipsi
IN contra
TCN ipsi
TCN contra
TCN relay
TCN comb
F
% of sustained RGCs
Sorted
Preference index
Sustained
Transient
TCN ipsi
TCN relay
IN ipsi
TCN comb
TCN contra
IN contra
IN mono
G
sustained
transient
***
% inputs
Inter-neurons
Thalamocortical neurons
H
ON-OFF DS
ON DS
JAM-B
none
***
% inputs
Inter-neurons
Thalamocortical neurons
I
≥ 1 DS or JAM-B
none
***
% cells
Inter-neurons
Thalamocortical neurons
J
ON
OFF
Preference index
IN ipsi
IN contra
TCN ipsi
TCN contra
K
Ipsi
Contra
Per cell
ON-OFF preference
Ipsi prevalence (%)
Sustained-Transient preference
Ipsi prevalence (%)

Supplemental Figure 2: Retinal inputs to dLGN interneurons are specialized

Related to Figure 2

A: Two close-by RGCs with arbor overlap can be classified as type 37 and type 6 based on the maximum z-projection, in which maxima are pseudo-colored according to their location in strata 1-10 (color bar). Scalebar: 50 µm.

B: Expected (lines) and measured (symbols) numbers of cell types plotted against the total number of classified presynaptic cells. Expected numbers were simulated as random draw with replacement (multinomial distribution) based on the weighted average of empirical distributions ('weighted TCN+IN p', blue) or the empirical distribution for interneurons ('IN p', red, Figure 2H). Thick line: mean, shaded area: mean ± standard deviation of the simulated numbers. Symbols indicate the measured numbers of RGC-types in contralateral or ipsilateral retinas presynaptic to the interneurons ('contra IN', 'ipsi IN' in red) or TCNs ('contra TCN', 'ipsi TCN' in gray) from which the retrograde tracing was initiated.

C, D: Specialization Z scores per cell for INs (red) and TCNs (gray), in (**D**) plotted against the total number of presynaptic RGCs.

E, F: Specialization Z scores (**E**), and preference indices (**F**) of individual retinal clusters grouped by the corresponding integration mode. INs (red), TCNs (gray). Mono: monocular, ipsi: ipsilateral, contra: contralateral, relay: monocular relay-mode, comb: monocular combination-mode. Relay- and combination-mode refer to the definition in Rompani et al.[S2]. Data groups in **F** are sorted along the x-axis by decreasing percentage of sustained RGCs found in the pooled data per group (upper panel).

G: Percentages of sustained and transient inputs to interneurons (red) and TCNs (gray[S2]). ***: $p<0.001$, Fisher's exact test.

H: Percentages of motion-selective (ON-OFF DS, ON DS and JAM-B) inputs to interneurons (red) and TCNs (gray[S2]). ***: $p<0.001$, Fisher's exact test, all three motion-selective against all other.

I: Proportion of interneurons (red) and TCNs (gray[S2]) receiving at least 1 DS or JAM-B input. ***: $p<0.001$, Fisher's exact test.

J: ON-OFF preference indices per retina for ipsilateral (ipsi) and contralateral (contra) retinas of INs (red) and TCNs (gray[S2]). $p=0.02$, Kruskal-Wallis test, $p=0.06$ for IN ipsi, posthoc sign-test with Bonferroni-Holm correction.

C, E, F, J: Horizontal lines indicate group averages, error bars indicate ± SEM.

K: ON-OFF preference indices (upper panel) and sustained-transient preference indices (lower panel) per retina and per cell for all binocular interneurons, plotted against ipsilateral prevalence (% of ipsilateral inputs from total). Preference indices are compared between ipsilateral (ipsi, yellow) and contralateral (contra, blue) retinas and indices per cell (black).

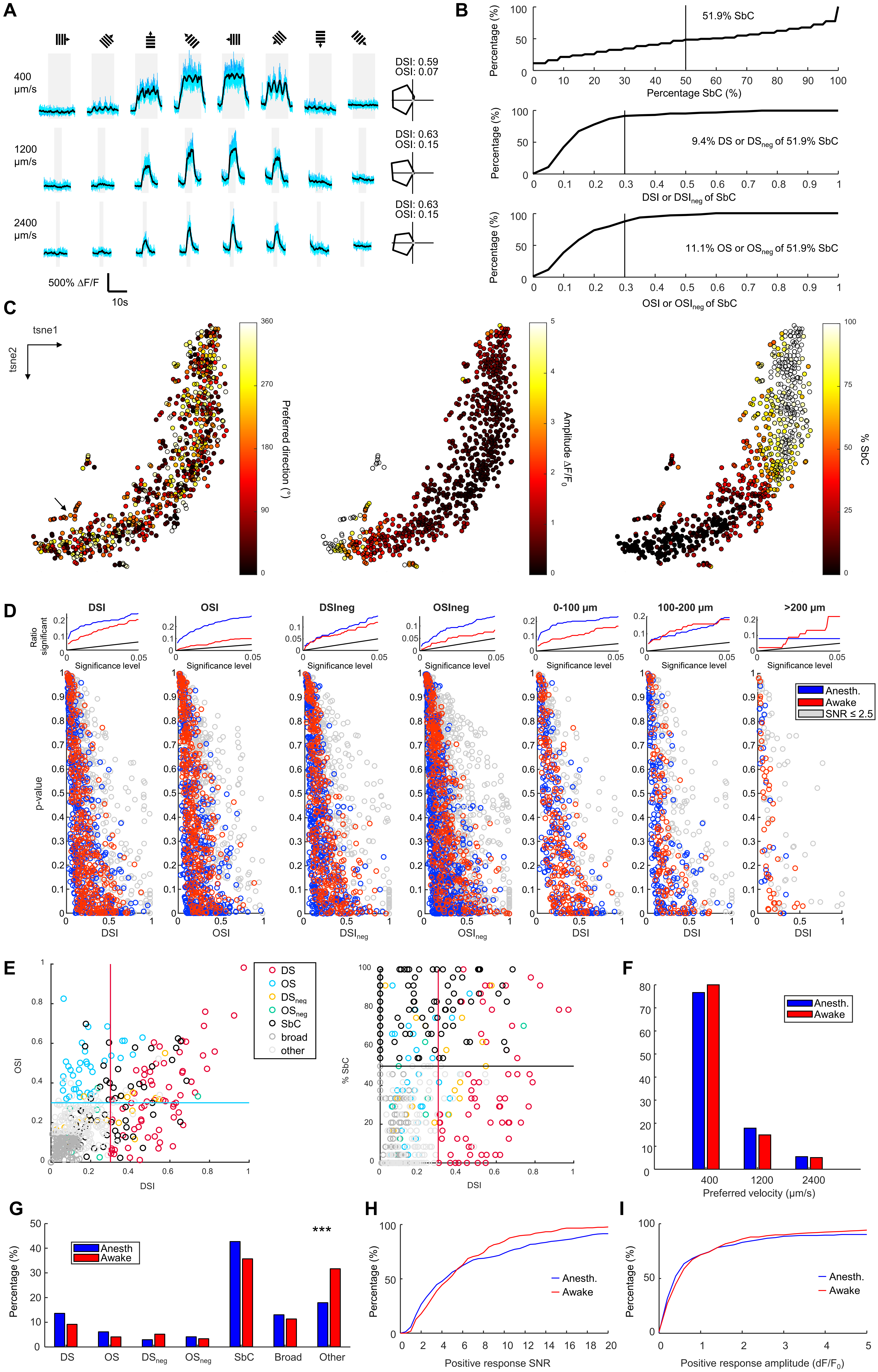
A
400 µm/s
1200 µm/s
2400 µm/s
DSI: 0.59
OSI: 0.07
DSI: 0.63
OSI: 0.15
DSI: 0.63
OSI: 0.15
500% ΔF/F
10s
B
51.9% SbC
Percentage SbC (%)
Percentage (%)
9.4% DS or DSneg of 51.9% SbC
DSI or DSIneg of SbC
11.1% OS or OSneg of 51.9% SbC
OSI or OSIneg of SbC
C
tsne1
tsne2
Preferred direction (°)
Amplitude ΔF/F0
% SbC
D
DSI
OSI
DSIneg
OSIneg
0-100 µm
100-200 µm
>200 µm
Ratio significant
Significance level
p-value
Anesth.
Awake
SNR ≤ 2.5
E
DS
OS
DSneg
OSneg
SbC
broad
other
OSI
DSI
% SbC
F
Anesth.
Awake
Preferred velocity (µm/s)
G
Anesth
Awake
***
Percentage (%)
DS
OS
DSneg
OSneg
SbC
Broad
Other
H
Anesth.
Awake
Positive response SNR
I
Anesth.
Awake
Positive response amplitude (dF/F0)

Supplemental Figure 3: dLGN interneurons display a wide range of visual features

Related to Figure 3

A: Example visual responses to the 24 stimuli (gratings drifting in 8 different directions with 3 different velocities). Blue: individual responses. Black: filtered median response. Top to bottom: 400, 1200, 2400 µm/s on the retina. Polar plots on the right display positive (black) and negative (gray, close to zero) response amplitudes plotted with respect to stimulus direction. DSI and OSI of the positive response amplitudes are annotated.

B: Cumulative histograms of suppressed-by-contrast response features. Upper panel: Cumulative histogram of the percentage of responses suppressed by contrast. Middle/lower panel: Cumulative histograms of the direction selectivity indices (middle, DSI and DSI_{neg}) and orientation selectivity indices (lower, OSI and OSI_{neg}) at the velocity that evoked largest absolute responses for interneurons with >50% suppressed-by-contrast responses.

C: t-SNE plots of the 48-dimensional response vectors (positive and negative responses to 24 stimuli) pseudo-colored for preferred direction of each cell in response to the speed which evoked the largest positive response (left), for maximum absolute amplitude $\Delta F/F_0$ (center), and for percentage of suppressed-by-contrast responses (right). Arrow on left panel points to a group of cells with anterior motion preference.

D: p-values for selectivity indices (lower panels) plotted against the corresponding selectivity indices for, from left to right: DSI, OSI, DSI_{neg}, OSI_{neg}, DSI for 0-100 µm, 100-200 µm and >200 µm, per cell for cells with SNR>2.5 of the positive (DSI, OSI) or negative (DSI_{neg}, OSI_{neg}) response in the anesthetized (blue) or awake (red) condition, as well as the remaining cells under both conditions (gray) with SNR≤2.5 of the positive (DSI, OSI) and negative (DSI_{neg}, OSI_{neg}) response. Upper panels: the ratio of cells with p-value below significance level for all cells with SNR>2.5 in the anesthetized (blue) or awake (red) condition, plotted against the significance level for the corresponding lower panels. Black line is the unity line, around which random noise responses would be located.

E: Left panel: OSI plotted against DSI for all interneurons recorded in anesthetized mice with positive or negative response with SNR>2.5. Right panel: Percentage of responses suppressed by contrast, plotted against DSI for all anesthetized data with positive or negative response with SNR>2.5. Unique response categories are annotated in color for each interneuron.

F: Distribution of stimulus velocity which evoked the largest absolute response under anesthetized (blue) or awake (red) condition.

G: Distribution of unique response categories under anesthetized (blue) or awake (red) condition. p<0.001, Chi-squared test; ***: p<0.001, posthoc Fisher's exact test for each category against all others with Bonferroni-Holm correction.

H: Cumulative histogram of SNR values of the positive responses under anesthetized (blue) or awake (red) condition. p=0.02, Kolmogorov-Smirnov test.

I: Cumulative histogram of positive response amplitudes $\Delta F/F_0$ under anesthetized (blue) or awake (red) condition. p=0.17, Kolmogorov-Smirnov test.

B-I: Interneurons recorded in 14 wild-type mice (**F-I** recorded at 0-100 µm depth).

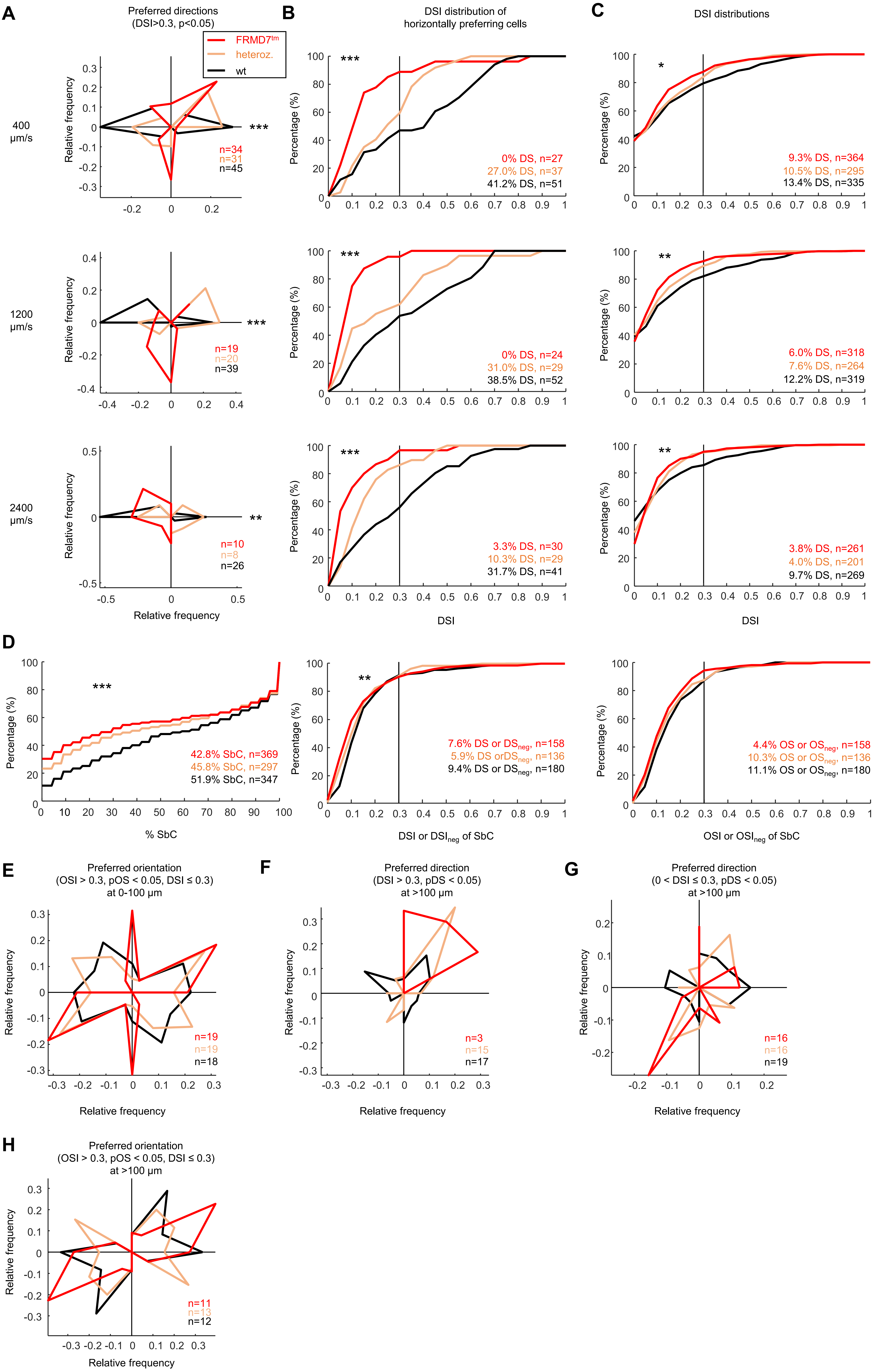
A
Preferred directions (DSI>0.3, p<0.05)
FRMD7tm
heteroz.
wt
400 μm/s
1200 μm/s
2400 μm/s
Relative frequency
***
n=34
n=31
n=45
***
n=19
n=20
n=39
**
n=10
n=8
n=26
B
DSI distribution of horizontally preferring cells
Percentage (%)
***
0% DS, n=27
27.0% DS, n=37
41.2% DS, n=51
0% DS, n=24
31.0% DS, n=29
38.5% DS, n=52
3.3% DS, n=30
10.3% DS, n=29
31.7% DS, n=41
DSI
C
DSI distributions
*
9.3% DS, n=364
10.5% DS, n=295
13.4% DS, n=335
**
6.0% DS, n=318
7.6% DS, n=264
12.2% DS, n=319
3.8% DS, n=261
4.0% DS, n=201
9.7% DS, n=269
D
42.8% SbC, n=369
45.8% SbC, n=297
51.9% SbC, n=347
% SbC
7.6% DS or DSneg, n=158
5.9% DS orDSneg, n=136
9.4% DS or DSneg, n=180
DSI or DSIneg of SbC
4.4% OS or OSneg, n=158
10.3% OS or OSneg, n=136
11.1% OS or OSneg, n=180
OSI or OSIneg of SbC
E
Preferred orientation (OSI > 0.3, pOS < 0.05, DSI ≤ 0.3) at 0-100 μm
n=19
n=19
n=18
F
Preferred direction (DSI > 0.3, pDS < 0.05) at >100 μm
n=3
n=15
n=17
G
Preferred direction (0 < DSI ≤ 0.3, pDS < 0.05) at >100 μm
n=16
n=16
n=19
H
Preferred orientation (OSI > 0.3, pOS < 0.05, DSI ≤ 0.3) at >100 μm
n=11
n=13
n=12

Supplemental Figure 4: Horizontal direction selectivity is inherited from the retina

Related to Figure 4

A: Polar plots of preferred directions of interneurons with DSI>0.3, pDS<0.05 in wild-type (black), hemizygous FRMD7^{tm} (red), and heterozygous FRMD7^{tm} (orange) mice. ***: p<0.001 (upper and middle), **: p=0.007 (bottom), Fisher's exact test for FRMD7^{tm} compared to wild-type mice with Bonferroni-Holm correction.

B: Cumulative histogram of DSI values across interneurons preferring horizontal motion (population vector direction 0 or 180° ± 15°) and with SNR>2.5 of the positive response in wild-type (black), hemizygous FRMD7^{tm} (red), and heterozygous FRMD7^{tm} (orange) mice. ***: p<0.001, Kolmogorov-Smirnov test for FRMD7^{tm} compared to wild-type mice with Bonferroni-Holm correction.

C: Cumulative histogram of DSI values across all interneurons in wild-type (black), hemizygous FRMD7^{tm} (red), and heterozygous FRMD7^{tm} (orange) mice. DSI was set to zero if the SNR of the positive response was ≤2.5. *: p=0.02, **: p<0.01, Kolmogorov-Smirnov test for FRMD7^{tm} compared to wild-type mice with Bonferroni-Holm correction.

D: Cumulative histogram of suppressed-by-contrast features in wild-type (black), hemizygous FRMD7^{tm} (red), and heterozygous FRMD7^{tm} (orange) mice. Left panel: Cumulative histogram of the percentage of responses suppressed by contrast. Middle/right panel: Cumulative histograms of positive or negative direction selectivity index (middle, DSI or DSI_{neg}) and orientation selectivity index (right, OSI or OSI_{neg}) for interneurons with >50% suppressed-by-contrast responses. The selectivity index of the largest absolute response is indicated. **: p=0.004, ***: p<0.001, Kolmogorov-Smirnov test with Bonferroni-Holm correction.

E: Polar plot of preferred orientations of orientation-selective, not DS interneurons (OSI>0.3, pOS<0.05, DSI≤0.3) in wild-type (wt, black), hemizygous FRMD7^{tm} (red) and heterozygous FRMD7^{tm} (orange) mice.

F: Polar plot of preferred directions of direction-selective interneurons (DSI>0.3, pDS<0.05) in wild-type (wt, black), hemizygous FRMD7^{tm} (red) and heterozygous FRMD7^{tm} (orange) mice recorded below 100 µm depth.

G: Polar plot of the preferred directions of interneurons with significant directional bias (0<DSI≤0.3, pDS<0.05) in wild-type (wt, black), hemizygous FRMD7^{tm} (red) and heterozygous FRMD7^{tm} (orange) mice recorded below 100 µm depth.

H: Polar plot of preferred orientations of orientation-selective, not DS interneurons (OSI>0.3, pOS<0.05, DSI≤0.3) in wild-type (wt, black), hemizygous FRMD7^{tm} (red) and heterozygous FRMD7^{tm} (orange) mice recorded below 100 µm depth.

A-E: Data from the upper 100 µm. **F-H**: Data from the lower 100 µm.

A-H: Interneurons, number annotated as n, recorded in 14 wild-type, 5 hemizygous FRMD7^{tm} und 3 heterozygous FRMD7^{tm} mice.

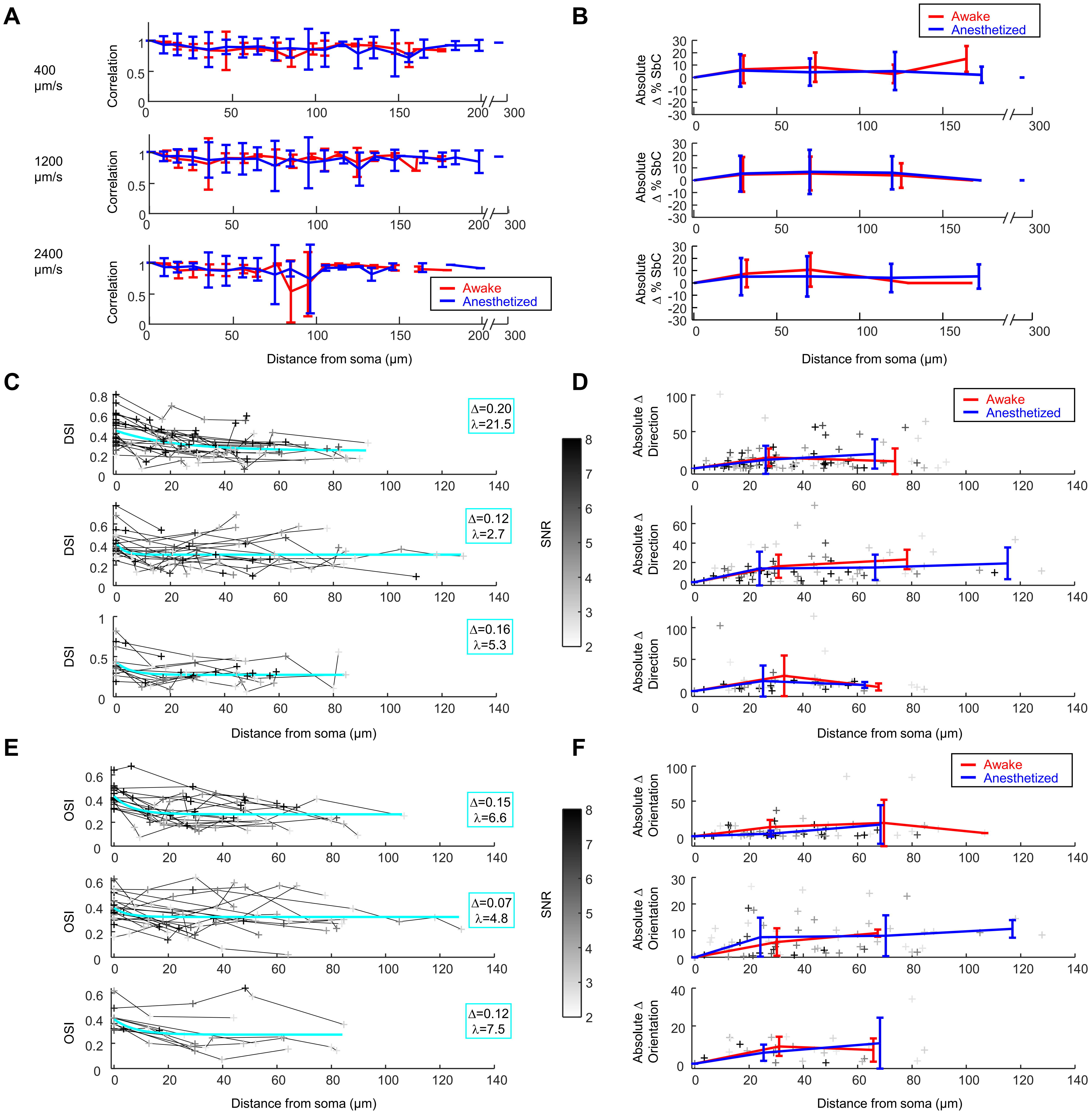
A
400 μm/s
1200 μm/s
2400 μm/s
Correlation
Awake
Anesthetized
Distance from soma (μm)
B
Absolute Δ % SbC
Awake
Anesthetized
Distance from soma (μm)
C
DSI
Δ=0.20
λ=21.5
Δ=0.12
λ=2.7
Δ=0.16
λ=5.3
SNR
Distance from soma (μm)
D
Absolute Δ Direction
Awake
Anesthetized
Distance from soma (μm)
E
OSI
Δ=0.15
λ=6.6
Δ=0.07
λ=4.8
Δ=0.12
λ=7.5
SNR
Distance from soma (μm)
F
Absolute Δ Orientation
Awake
Anesthetized
Distance from soma (μm)

Supplemental Figure 5: Somatic visual features extend into the dendrites

Related to Figure 6

A: Correlation of the 16-dimensional response vectors (positive and negative response amplitudes to 8 directions) with their respective somatic response vector, plotted against Euclidean distance from the soma. Data averaged over 10 µm bins. Blue: anesthetized, red: awake mice. Here and in all subsequent plots of **A**-**F**: Top: 400 µm/s; middle: 1200 µm/s; bottom: 2400 µm/s stimulus velocity.

B: Absolute difference in the percentage of suppressed-by-contrast responses compared to the somatic responses, plotted against Euclidean distance from the soma.

C: DSI values of individual compartments, plotted against Euclidean distance from the soma.

D: Absolute difference in the preferred direction compared to the somatic response for data in **C**, plotted against Euclidean distance from the soma

E: OSI values of individual compartments, plotted against Euclidean distance from the soma.

F: Absolute difference in the preferred orientation compared to the somatic response for data in **E**, plotted against Euclidean distance from the soma

C, D: Only cells with at least one compartment with DSI>0.3 included. **E, F**: Only cells with at least one compartment with OSI>0.3 included. **C, E**: Cyan: mono-exponential fit with length constant λ and amplitude Δ as indicated ($y=\Delta \cdot \exp(-x/\lambda) + y_0$).

A, B, D, F: Red/blue errorbars: mean ± standard deviation of data recorded under awake (red) or anesthetized (blue) conditions in 50 µm bins. Gray scale: SNR.

A-F: Interneurons recorded in 5 wild-type, 5 hemizygous FRMD7^{tm} und 3 heterozygous FRMD7^{tm} mice.

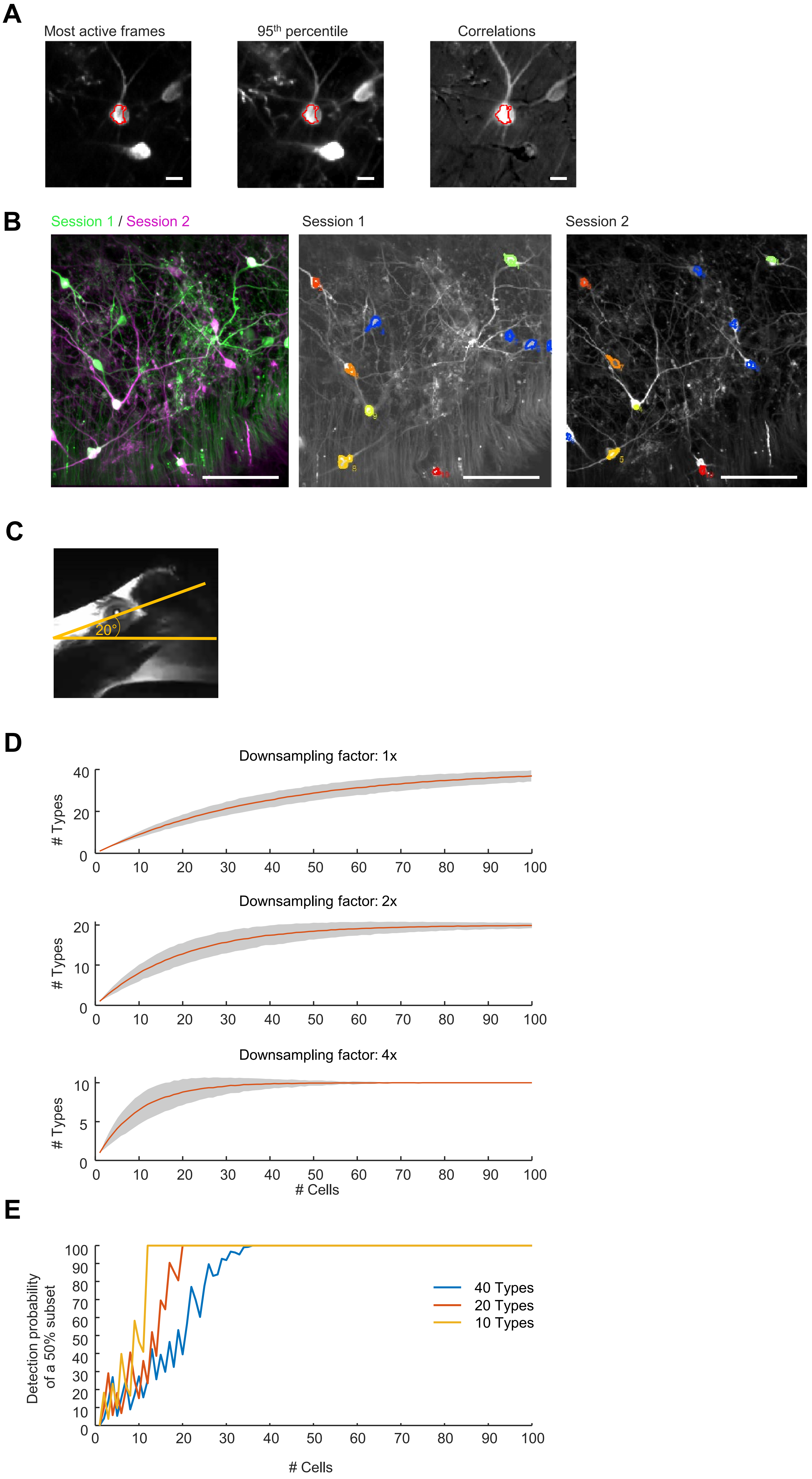
A
Most active frames
95th percentile
Correlations
B
Session 1 / Session 2
Session 1
Session 2
C
20°
D
Downsampling factor: 1x
Types
40
20
0
0 10 20 30 40 50 60 70 80 90 100
Downsampling factor: 2x
Types
20
10
0
0 10 20 30 40 50 60 70 80 90 100
Downsampling factor: 4x
Types
10
5
0
0 10 20 30 40 50 60 70 80 90 100
Cells
E
Detection probability of a 50% subset
100 90 80 70 60 50 40 30 20 10 0
0 10 20 30 40 50 60 70 80 90 100
Cells
40 Types
20 Types
10 Types

Supplemental Figure 6: Method details

Related to STAR Methods

A: ***Identification of interneuron somata*****:** After selecting a region of interest (ROI, red) with putative somata, the projection over the 500 most active frames (left panel), local 95% percentile projection (middle panel), and local correlation matrix (right panel) were inspected to confirm that the ROI corresponds to a soma. Scale bar: 10 µm.

B: ***Avoiding duplication of cells*****:** Recordings from the same or overlapping regions were aligned (left panel) and ROIs belonging to the same cells identified (right panels, ROIs belonging to the same cell plotted in the same color, non-matching ROIs in blue). Scale bar: 100 µm.

C: ***Correction for head-mounting angle*****:** The angle between the horizontal axis connecting the two eye corners and the table on which the stimulation screen was placed was determined. All stimulus directions were adjusted accordingly such that horizontal motion refers to motion along the horizontal axis of the eye.

D: ***Simulations how the number of cell-types influences the specialization Z scores:*** In a model simulation with 40 equi-distributed cell types, cell-types were downsampled by a factor of 4 (lower panel), 2 (middle panel) or not downsampled (upper panel). The expected number of cell types is plotted against the number of classified cells. Orange: expected value, gray: ± 1 standard deviation.

E: ***Pooling cell-types meaningfully increases the power to detect specialization:*** The probability for detecting a specialized subset consisting of 50% of the cell types plotted against number of classified cells, for a classification with 40 types (no downsampling, blue), 20 types (2x downsampling, orange), or 10 types (4x downsampling, yellow), assuming that cell-types from the specialized subset are only pooled with each other.

SUPPLEMENTAL REFERENCES

[S1] Franklin, K.B.J., and Paxinos, G. (2007). The mouse brain in stereotaxic coordinates 3rd ed. (Academic press).

[S2] Rompani, S.B., Müllner, F.E., Wanner, A., Zhang, C., Roth, C.N., Yonehara, K., and Roska, B. (2017). Different Modes of Visual Integration in the Lateral Geniculate Nucleus Revealed by Single-Cell-Initiated Transsynaptic Tracing. Neuron *93*, 767-776.e6. 10.1016/j.neuron.2017.01.028.
